# Genetic prediction of antihyperglycemic drug targets and risk of epilepsy: a mendelian randomisation study

**DOI:** 10.1186/s40360-023-00718-2

**Published:** 2024-01-02

**Authors:** Kaiping Zhou, Huan Yang, Zhihao Xie, Weiping Wang, Zhenzhen Qu

**Affiliations:** 1 Key Laboratory of Neurology of Hebei Province, Department of Neurology, The Second Hospital of Hebei Medical University, Shijiazhuang, China; 2 The Second Hospital of Jilin University, Changchun, China

**Keywords:** Antihyperglycemic Drugs, Target genes, Epilepsy, Mendelian randomisation

## Abstract

A connection between diabetes and an increased risk of epilepsy has been suggested by observational studies. Animal studies have also shown that antihyperglycemic drugs can improve seizures. However, it is unclear whether antihyperglycemic drugs have a causal role in epilepsy in humans. To investigate this potential causal relationship, a Mendelian randomisation study was conducted using International League Against Epilepsy data as the discovery set and FinnGen data as the replication set. It was discovered that three antidiabetic drug target genes, ETFDH, CYP21A2 and CYP2D6, were involved in the occurrence of epilepsy. In particular, ETFDH was identified as a target gene in both the discovery set (inverse variance weighting [IVW], odds ratio [OR] = 1.018, 95% confidence interval [CI], 1.004–1.033, p = 0.009) and replication set (IVW, OR = 1.074, 95% CI, 1.034–1.114, p = 0.00016), and CYP21A2 was identified in the discovery set (IVW, OR = 1.029, 95% CI, 1.005–1.053, p = 0.016) and replication set (IVW, OR = 1.057, 95% CI, 1.001–1.116, p = 0.045) as having a causal association with an increased risk of epilepsy. Conversely, the CYP2D6 gene was found to be a protective factor for epilepsy in both the discovery set (IVW, OR = 0.0984, 95% CI, 0.969–0.998, p = 0.025) and replication set (IVW, OR = 0.977, 95% CI, 0.955–1.000, p = 0.046). A search of DrugBank revealed that metformin, an anti-glucose drug, is an inhibitor of the ETFDH gene and may have a potential therapeutic effect on epilepsy.

**Supplementary Information:**

The online version contains supplementary material available at 10.1186/s40360-023-00718-2.

## Introduction

Epilepsy is a complex condition affecting over 70 million people worldwide. It has multiple risk factors and a strong genetic predisposition rather than a single clinical presentation and cause [1]. Its most common treatment methods are drug therapy, surgery, nerve stimulation and diet modifications [2]. The majority of patients with epilepsy choose antiepileptic drugs to control seizures based on the assumption that the side effects will not interfere with everyday life. However, one-third of patients with epilepsy do not achieve complete seizure control [3]. Long-term seizures cause cognitive impairment, anxiety, depression and other epilepsy-related complications [4]. Since developing new drugs is time-consuming and expensive, reusing old drugs to treat common and rare diseases has gradually become a new trend called drug reuse, also known as drug repositioning. The term refers to the discovery of new uses for drugs outside the original clinical indications [5].

In epidemiologic studies, diabetes has been linked to an increased risk of epileptic seizures [6–9]. Animal studies have shown some antihyperglycemic drugs, such as liraglutide, sitagliptin, rosiglitazone and metformin, to be effective in reducing seizures and improving cognitive impairment [10–14]. Randomised controlled clinical trials are the gold standard for determining drug efficacy; however, due to ethical reasons, long follow-up time, high cost and other factors, there is a lack of high-quality, large-scale randomised controlled trials and studies on the effect of antihyperglycemic drugs on seizures in the population. Therefore, Mendelian randomisation (MR) was developed as a new research method. It estimates the causal relationship between exposure and outcome using genetic variants as instrumental variables (IVs). Exposure in this context refers to any factor of interest that may influence the outcome, such as modifiable lifestyles and biomarkers [15]. As such, MR can be used to assess the causal relationship between drug target genes and diseases. Mendelian randomisation maximisation is considered superior to observational studies due to its avoidance of endogenous problems in regression analysis, such as reverse causality, confounding factors and measurement error [16].

Mendelian randomisation analysis has been employed to investigate the potential connections between various drug treatments and certain diseases, such as antihypertensive drugs and psychiatric disorders [17], antihyperglycemic drugs and Parkinson’s disease [18] and the risk of lipid-lowering drugs, antihyperglycemic drugs and Alzheimer’s disease [19, 20]. In this study, the Genotype-Tissue Expression Project (GTEx)-V8 database was utilised to verify the proxy antihyperglycemic drug target genes of IVs, and a two-sample MR analysis was conducted to explore the effects of different antihyperglycemic drug treatments on epilepsy.

## Materials and methods

### Study design

**Fig. 1:**
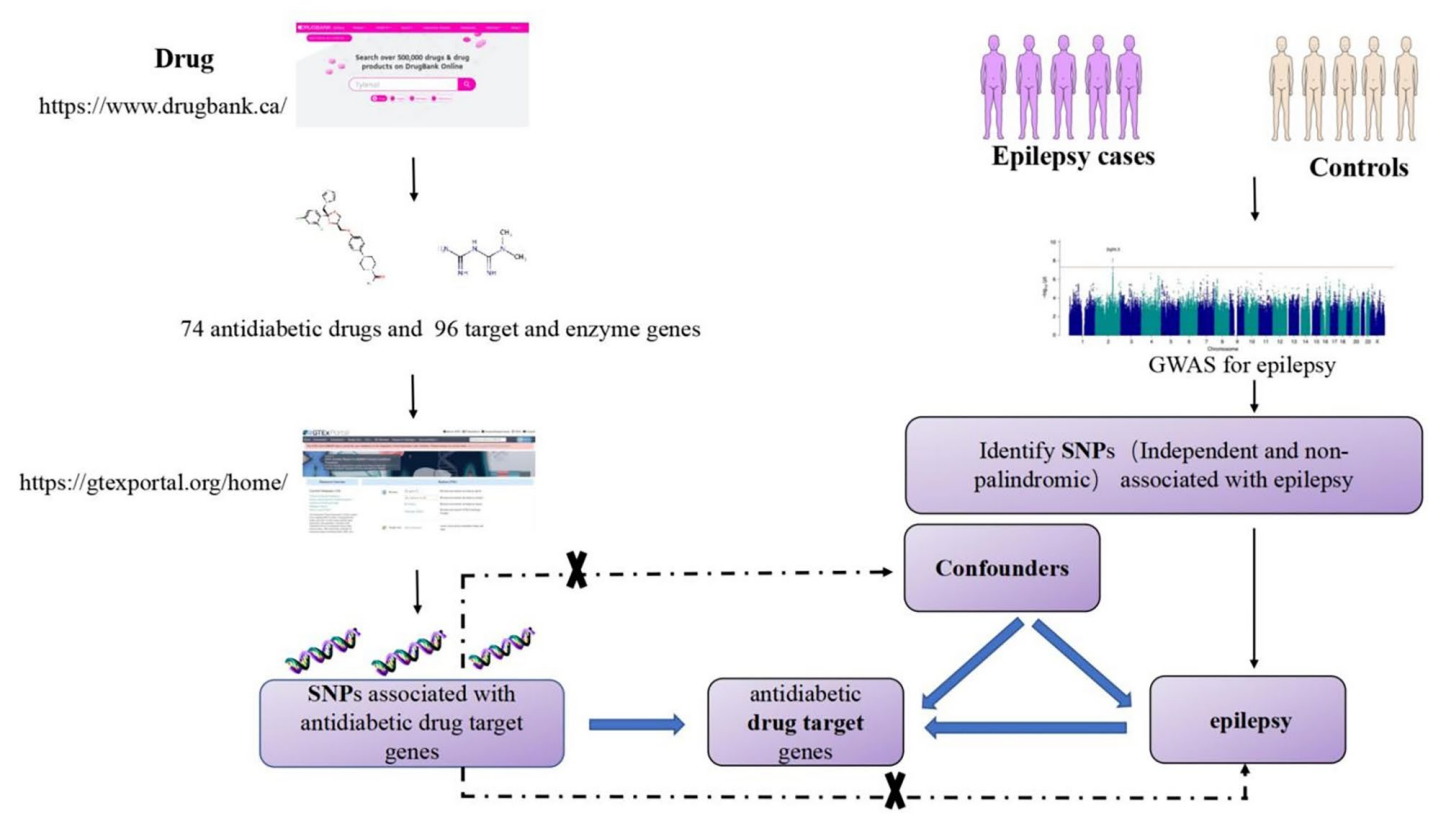
Schematic representation of this study

The present two-sample MR study was conducted with the aim of exploring the potential relationship between antihyperglycemic drugs and epilepsy. The study focuses on 96 gene targets of 74 diabetes medications, including metformin, glyburide, gliclazide, acarbose, miglitol, pioglitazone, repaglinide, sitagliptin, dapagliflozin and others. The discovery set was based on data from the International League Against Epilepsy (ILAE), whereas the replication set was derived from the FinnGen consortium data. The intersection of the two datasets was used to determine the relationship between antihyperglycemic drugs and epilepsy and to identify a new pathway for the treatment of epilepsy (Fig. 1). Since this study utilised existing summary genome-wide association study data, separate ethical approval was not required, as all previous studies had already obtained ethical approval in accordance with the Declaration of Helsinki.

### Data sources and instrumental variable selection

The anti-glucose drug targets in the DrugBank database (http://www.drugbank.ca/) were searched, and it was found that 74 drugs or compounds that have been used in clinical practice but not yet been used in clinical trials were under investigation for the treatment of diabetes. The database yielded a total of 96 target genes for all antihyperglycemic drugs. The GTEx-V8 database (https://gtexportal.org/home/), which studied the tissue specificity of gene expression and regulation using nearly 1,000 people in 54 lesion tissue samples, was used to search single nucleotide polymorphisms (SNPs) associated with drug target genes. The brain tissues included were brain_amygdala, brain_anterior_cingulate_cortex, brain_caudate_basal_ganglia, brain_cerebellar_hemisphere, brain_cerebellum, brain_cortex, brain_frontal_cortex, brain_hippocampus, brain_hypothalamus, brain_nucleus_accumbens_basal_ganglia, brain_putamen_basal_ganglia, brain_spinal_cord_cervical and brain_substantia_nigra.

Quantitative trait locus (QTL) can be understood as quantitatively related gene loci. Expression quantitative trait loci (eQTL) refer to gene expression trait loci. Single nucleotide polymorphisms expression quantitative trait loci can be IVs in the drug-target MR analysis. Each SNP used as an IV met the following criteria: SNPs(eQTLs) associated with antihyperglycemic drug targets using the data from the GTEx-V8 database’s brain tissues. A p value cut-off of 0.05 was used to select the genetic variants associated with the expression levels of the 96 genes (defined by the distribution-adjusted empirical p values using a false discovery rate threshold of 0.05; see http://www.gtex-portal.org/home/documentation for details). A series of quality control steps was implemented to select eligible IVs. First, the cut-off for minor allele frequency was set to > 0.01 and < 0.99. Second, referring to the criteria for processing the eQTLs data in the GTEx-V8, these do not include synonymous SNPs. Third, the linkage disequilibrium threshold for clumping was set to r2 = 0.3, and the clumping window size was set to 500 kB. Finally, the SNPs with inconsistent alleles between the exposure and outcome samples and palindromic A/T or G/C alleles were excluded. Supplementary Table 1 lists the names of the 74 drugs and the 96 drug target genes.

The ILAE provided data for the discovery set from a large genome-wide association study of epilepsy (15,212 cases and 29,677 controls) [21]. Establishing a replication set is of great significance for quality control in an MR study [22]. Furthermore, for the replication set, data from the FinnGen consortium (6,260 cases and 176,107 controls), which is publicly available with FinnGen Data Freeze 6 and includes 260,405 participants, was used. There were 16,962,023 variants and 2,861 endpoints (http://r6.finngen.fi/). The FinnGen consortium’s 6,260 cases were defined using the International Classification of Diseases-10 code G40.

### Statistical methods

Two-sample MR data were analysed using the TwoSample MR R package, version 0.5.6. The inverse variance weighting (IVW) method was used for the main analysis. Since the number of SNPs identified for each drug was relatively small, a Wald ratio analysis was added to the main analysis to estimate the causal effect of anti-glucose drug targets on epilepsy as a single working variable. Furthermore, various analysis methods, such as weighted median, weighted mode and MR–Egger, were used to strengthen the causal inference. In the sensitivity analysis, Cochran’s Q test was used to test for heterogeneity, and the intercept term of the MR–Egger method and the R package Mendelian Randomization Pleiotropy RESidual Sum and Outlier were used to test for multiple validity and a leave-one-out sensitivity analysis was performed. The statistical validity of MR was determined using the power calculations on the Mendelian Randomization website (https://shiny.cnsgenomics.com/mRnd/), where the F statistic represents the strength of the IV and can be calculated using the following formula: $$ F= {R}^{2}(N - 2)(1-{R}^{2})$$. The Bonferroni procedure was utilised to minimise the possibility of a type I error resulting from repeated calculations within the same datasets and adjust the significance threshold. A type I error is also known as a false positive error. It means that there is no overall difference, although the calculation results show differences, resulting in false positive results. According to this method, the adjusted test level was set at 0.00052 (0.05/96). A p value of < 0.00052 indicated a strong level of significance, whereas p values of 0.00052–0.05 suggested significant results.

## Results

### International league against epilepsy: the relationship between antihyperglycemic drug targets and epilepsy

**Fig. 2:**
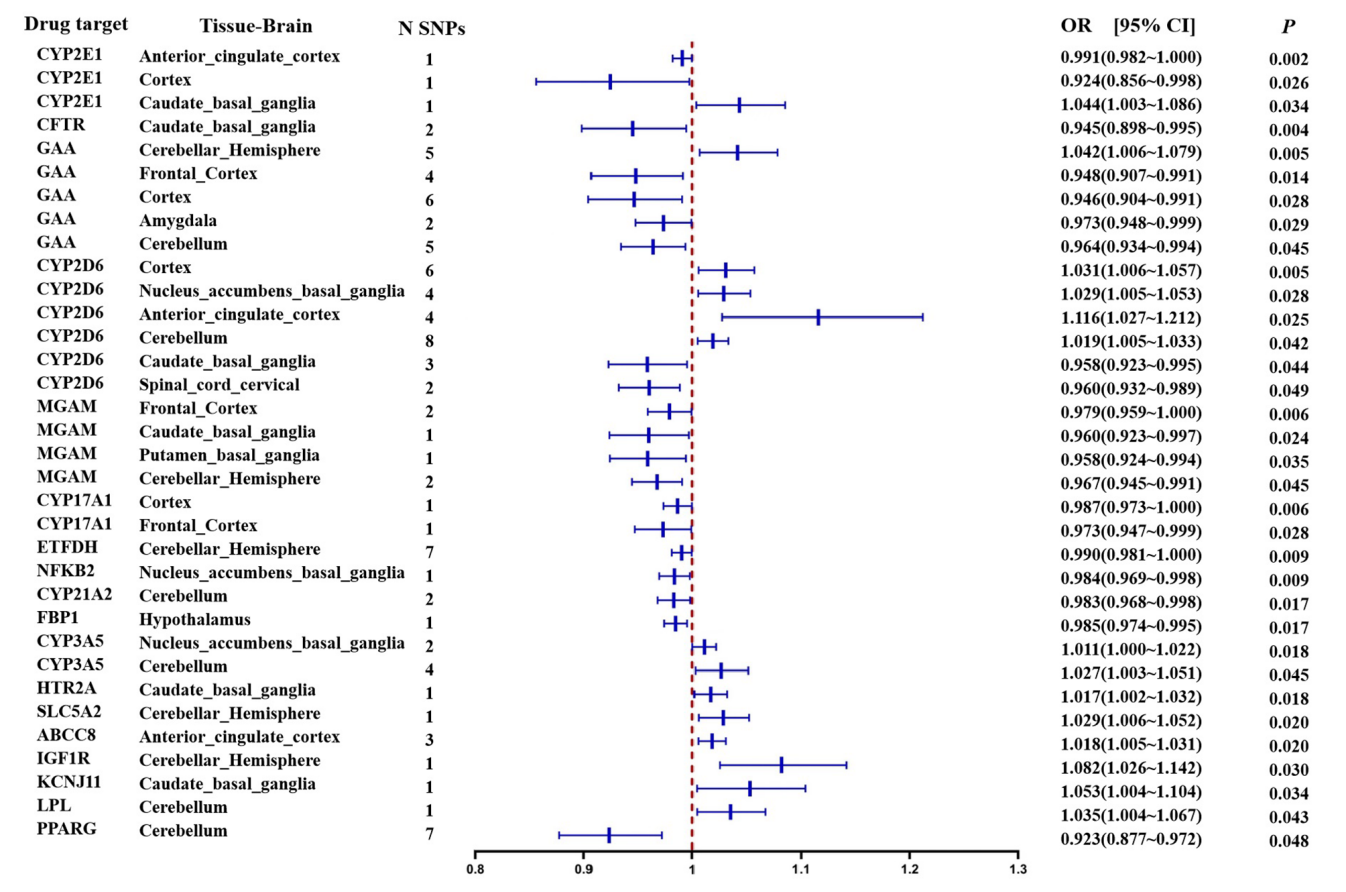
The odds ratios for genetically predicted antihyperglycemic drug targets associated with epilepsy in the International League Against Epilepsy

The Bonferroni correction criteria in the present study were too strict to identify any estimates that withstood the correction. However, out of a total of 96 calculations, 18 results demonstrated suggestive causal associations between antihyperglycemic drug targets and epilepsy, with IVW-derived p values of < 0.05. These 18 antihyperglycemic drug targets include CYP2E1, CFTR, GAA, CYP2D6, MGAM, CYP17A1, (ETFDH), NFKB2, CYP21A2, FBP1, CYP3A5, HTR2A, SLC5A2, ABCC8, IGF1R, KCNJ11, LPL and PPARG. Figure 2 illustrates these findings, and Supplementary Table 2 provides the detailed results. Additionally, Supplementary Figs. 1–7 present scatter plots, funnel plots, forest plots and analysis plots of one-by-one exclusion tests for SNPs with a value of ≥ 5. Scatter plot: The scatter plot’s ordinate shows how the SNP affected the exposure (anti-glucose drug target), and the abscissa axis shows how it affected the outcome (epilepsy). Each point on the plot shows an IV, and the grey line at each point shows the 95% confidence interval (CI). The coloured line shows the MR fitting results. Forest plot: The red line at the bottom of the forest plot, according to the IVW methodology, represents the causal influence of exposure (anti-glucose medication target) on the outcome (epilepsy). Funnel plot: A single IV exerted a slight pleiotropic effect on the estimated causal effects. Analysis plot of one-by-one exclusion tests: The bottom red line demonstrates that the overall error line does not change much after eliminating each SNP, indicating that the results are reliable.

### Further exploration of the relationship between antihyperglycemic drugs and epilepsy in the FinnGen consortium

**Fig. 3:**
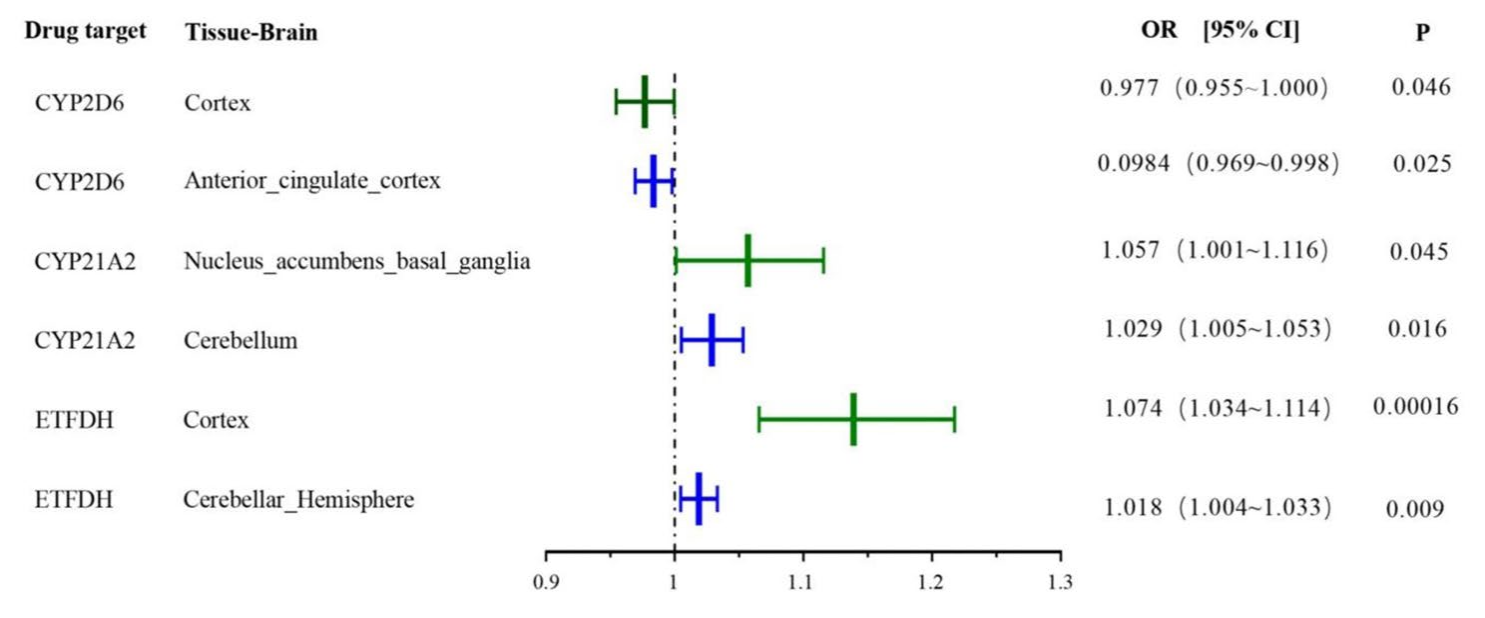
Associations of genetic proxies for antihyperglycemic drug targets in the International League Against Epilepsy and FinnGen. Blue, the International League Against Epilepsy, and Green, FinnGen.

**Fig. 4:**
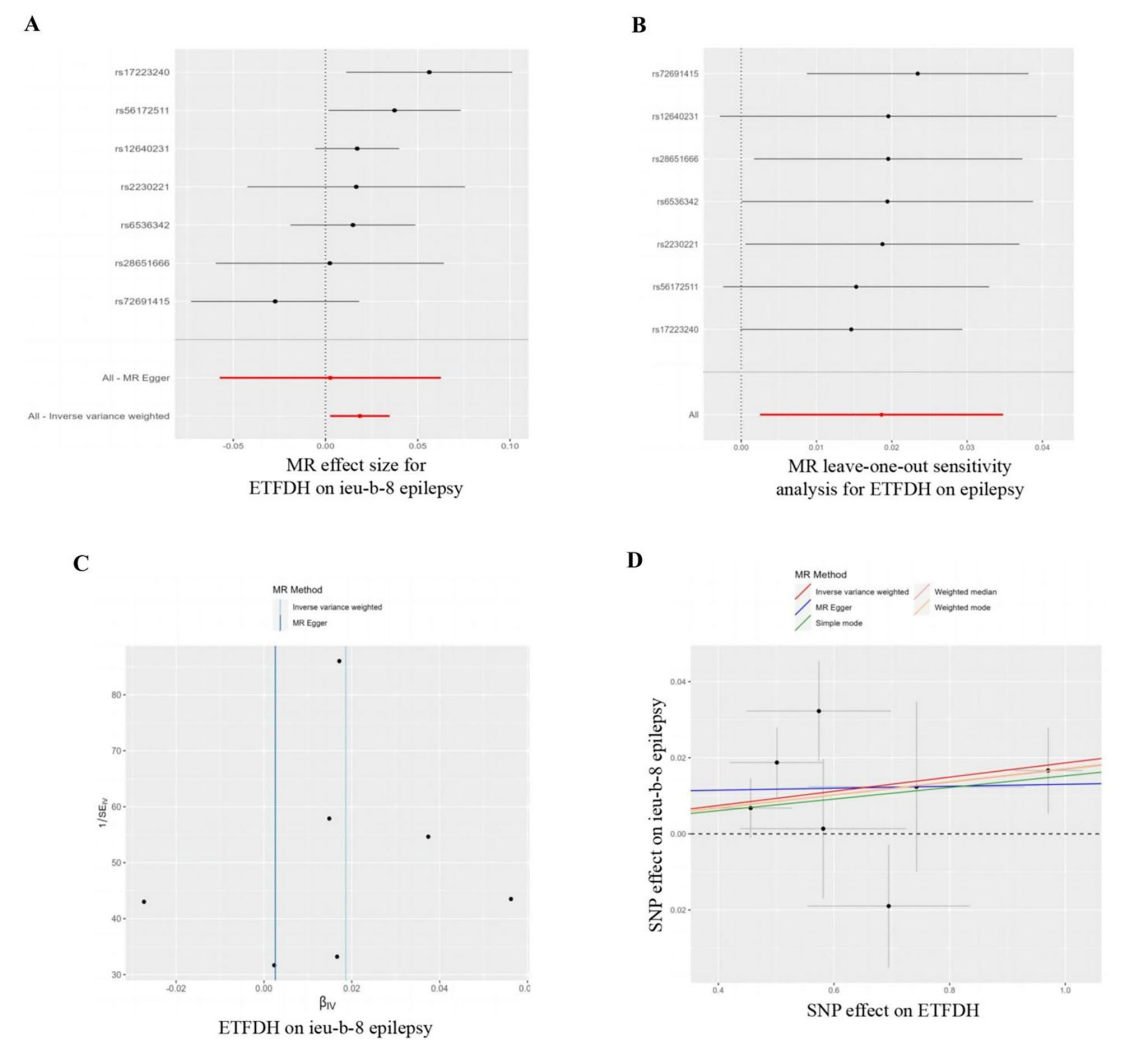
Mendelian randomisation assessment of the ETFDH expression in brain_cerebellar_hemisphere and epilepsy risk in the International League Against Epilepsy. (**A**) Forest plot. Each horizontal solid line reflects the result estimated for a single nucleotide polymorphism using the Wald ratio method. The bottom red line reflects the risk relationship between ETFDH and epilepsy under the IVW approach. (**B**) Leave-one-out analysis of genetic proxy ETFDH on epilepsy risk. (**C**) Funnel plot. Vertical lines show causal estimates using each of the two different methods to combine all single nucleotide polymorphisms into a single instrument. (**D**) Scatter plot. The slope of the straight line corresponds with the causal estimates using the five different methods

**Fig. 5:**
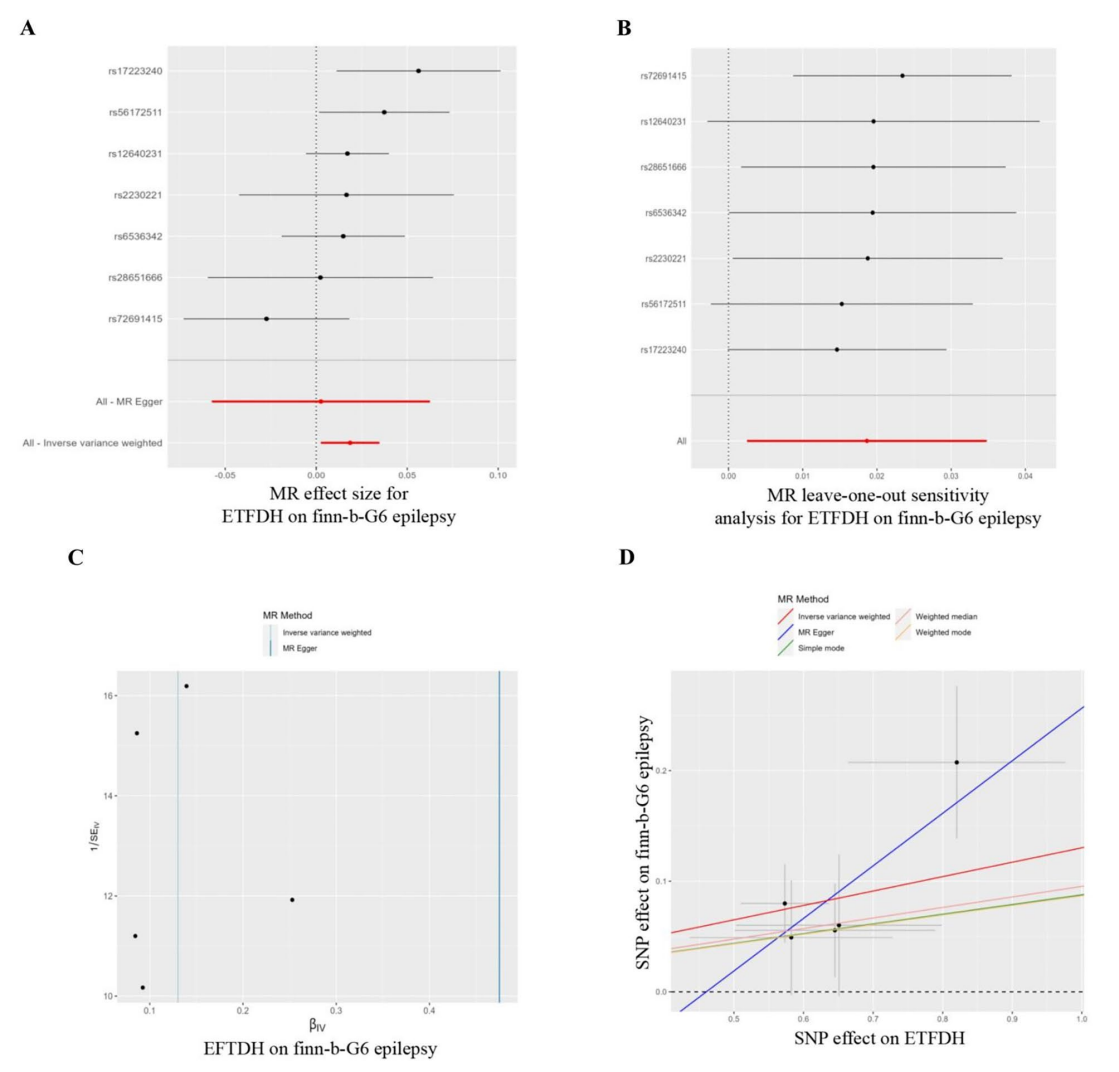
Mendelian randomisation assessment of the ETFDH expression in brain_cortex and epilepsy risk in FinnGen. (see the legend on the previous Figure). (**A**) Mendelian randomisation effect size for ETFDH on finn-b-G6 epilepsy. (**B**) Mendelian randomisation leave-one-out sensitivity analysis for ETFDH on finn-b-G6 epilepsy. (**C**) EFTDH on finn-b-G6 epilepsy. (**D**) Single nucleotide polymorphism effect on ETFDH.

Three antihyperglycemic drug targets were validated in the FinnGen consortium data and were consistent with the preliminary analysis results: ETFDH, CYP21A2 and CYP2D6 (Table 1; Fig. 3). The ETFDH (expression in the cerebellar hemispheres) were predicted by the gene in the discovery set (IVW, odds ratio [OR] = 1.018, 95% CI = 1.004–1.033, p = 0.009, Fig. 4). In the replication set, ETFDH was expressed in the cortex (IVW, OR = 1.074, 95% CI = 1.034–1.114, p = 0.00016, Fig. 5). Meanwhile, CYP21A2 (expression in the cerebellum) was predicted by the gene in the discovery set (IVW, OR = 1.029, 95% CI = 1.005–1.053, p = 0.016, Supplementary Fig. 8). In the replication set, CYP21A2 was expressed in the brain_nucleus_accumbens_basal_ganglia (IVW, OR = 1.057, 95% CI = 1.001–1.116, p = 0.045, Supplementary Fig. 9). The findings of this study suggest a causal relationship between an increased risk of epilepsy and several antihyperglycemic drug targets. Meanwhile, CYP2D6 may act as a protective factor for epilepsy. Specifically, CYP2D6 was found to be expressed in the brain_anterior_cingulate_cortex in the discovery set (IVW, OR = 0.0984, 95% CI = 0.969–0.998, p = 0.025, Supplementary Fig. 10) and the cortex in the replication set (IVW, OR = 0.977, 95% CI = 0.955–1.000, p = 0.046 in Supplementary Fig. 11). All three antihyperglycemic targets exhibited strong instrumentation, with F-statistic values exceeding the common threshold of 10. Moreover, there was minimal heterogeneity among the three targets in the heterogeneity test, and the sensitivity analysis demonstrated the stability of the causal effect (Table 1).

**Table 1:** MR Analysis of anti-glucose drug target genes and epilepsy

Stage	Target gene	Method	Nsnp	Beta	Pval	Q	Ple	R2	F	Drugs and Pharmacological action
Discovery	ETFDH	Inverse variance weighted (fixed effects)	7	0.019	0.0091	0.24	0.61	0.68	19.10	Metformin (Yes inhibitor)
Replication	ETFDH	Inverse variance weighted (fixed effects) Weighted median	5 5	0.130 0.095	0.0001 0.0360	0.55	0.33	0.23	12.20	
Discovery	CYP21A2	Inverse variance weighted	2	0.029	0.0166	NA	NA	0.19	20.17	Levoketoconazole (No)
Replication	CYP21A2	Inverse variance weighted (fixed effects)	5	0.055	0.0451	0.07	0.24	0.24	25.92	
Discovery	CYP2D6	Inverse variance weighted (fixed effects) Weighted median	4 4	-0.017 -0.019	0.0253 0.0324	0.78	0.56	0.54	27.60	Dapagliflozin (No) Phenformin (Unkonw)
Replication	CYP2D6	Inverse variance weighted (fixed effects)	13	-0.023	0.0460	0.69	0.78	0.90	12.56	Nateglinide (Unkonw) Rosiglitazone (Unkonw) Alogliptin (Unkonw)

Note: Stage refers to our MR Research stage, which is divided into discovery set and validation set. method refers to the meta method we used; Nsnp refers to the number of instrumental variables used; Beta refers to the effect size in the MR Analysis; and P-val refers to the statistical significance of the MR Analysis. Q refers to the significance of the heterogeneity test and Ple refers to the significance level of the pleiotropy test in the MR Analysis. R2 refers to the degree to which the instrumental variable explains the exposure. F is the F-test statistic. Drugs and Pharmacological action refer to the drugs related to the target genes of anti-glucose drugs and the pharmacological effects between them

## Discussion

**Fig. 6:**
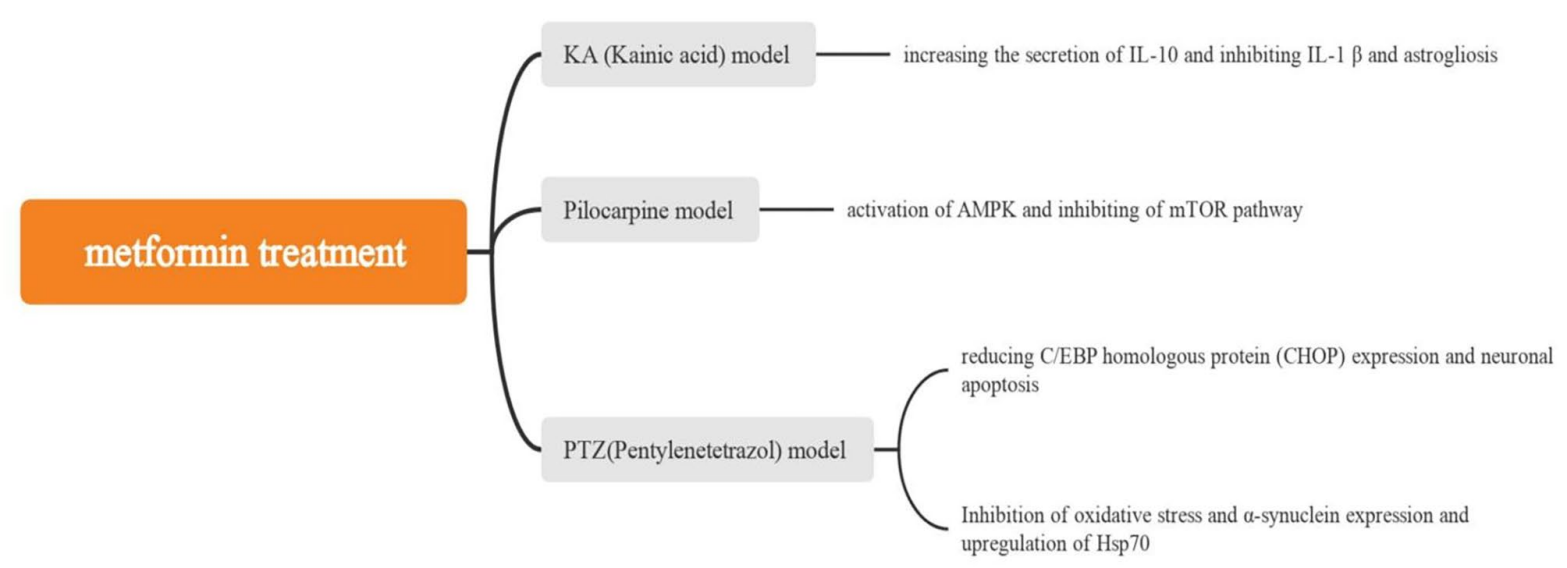
Mechanisms by which metformin improves seizures in animal models

This study represents the first use of MR analysis to investigate the effect of antihyperglycemic drugs on epilepsy. The study identified three anti-glucose drug target genes, ETFDH, CYP21A2 and CYP2D6, associated with epilepsy. A search in DrugBank for drugs related to the target genes of anti-glucose drugs and the pharmacological effects of binding drugs revealed that metformin is related to the ETFDH gene (Table 1). Furthermore, as an inhibitor of the ETFDH gene, metformin has the potential to be used as a therapeutic treatment for epilepsy. Previous studies have shown that ETFDH is a potential target for ageing and Alzheimer’s disease [23, 24]. Meanwhile, the results of this study reveal that ETFDH is a potential target for anti-epilepsy. Two clinical trials have shown the potential role of metformin in the treatment of epilepsy. One trial involved 12 patients with Lafora disease who were treated with metformin and were found to experience a slower progression of the disease [25]. The second trial was a multicentre, randomised, double-blind, placebo-controlled clinical trial of metformin in the treatment of tuberous sclerosis, which found that metformin reduced seizure frequency compared with the placebo [26]. From a clinical practice perspective, the present findings, together with previous randomised controlled trials, provide some support for the potential therapeutic role of metformin in patients with epilepsy. In this MR study, medication adherence and confounding factors were less of a concern, as the genetically instrumented exposure is lifelong. The preliminary study of DrugBank data and the results in this paper indicate that ETFDH, the target of metformin, is associated with an elevated risk of epilepsy and that metformin has a potential therapeutic value for epilepsy, although the specific mechanism still needs to be further explored. At present, although the mechanism by which metformin improves epilepsy is not fully understood, a growing number of animal studies have shown that metformin can improve seizures in various ways. For example, in a mouse model of kainic acid epilepsy, Somayeh et al. found that metformin increased interleukin (IL)-10 secretion and inhibited IL-1β and astrocyte regeneration, achieving anti-inflammatory effects, with metformin potentially exerting at least some of its anti-inflammatory effects by increasing the progranulin level [27]. Soraya et al. found that metformin activated the adenosine monophosphate-activated protein kinase signalling pathway and decreased the mammalian target of rapamycin (mTOR) expression in a pilocarpine epilepsy rat model [28]. Jing et al. revealed that the C/EBP homologous protein pathway expression and apoptosis induced by status epilepticus in rats were reduced with the use of metformin [29]. The antiepileptic and neuroprotective effects of metformin in Pentetrazol-induced epilepsy may be due to the inhibition of apoptosis, attenuation of oxidative stress and α-synuclein expression and upregulation of Hsp70 [30] (Fig. 6). Metformin has also been shown to have antiepileptic effects in worms, zebrafish and fly epilepsy animal models [22, 31]. Although metformin has been shown to have therapeutic effects in various animal models of epilepsy, few randomised controlled trials have been conducted to detect its potential effects in the clinical treatment of epilepsy. However, the present findings may support the potential therapeutic effect of metformin therapy in patients with epilepsy and is a promising candidate for the treatment of human epilepsy. Further research is needed to determine its efficacy and safety.

The present study has limitations. First, data from European populations was used to avoid racial confusion, and the experimental results require confirmation across other ethnic populations. Second, the MR analysis of genetic variation as an IV more accurately reflects the long-term effect of antihyperglycemic drug target genes on epilepsy, whereas the effect of short-term drug treatment on epilepsy cannot be inferred. Third, constraints in data availability prevented subgroup analyses from being made based on age and sex, and potential sample overlap between the two datasets could not be accounted for, potentially leading to bias in the overall estimates. Fourth, in the present study, the MR estimates were adjusted for multiple testing. The 18 antihyperglycemic drug targets showed a causal association (0.00052 < p < 0.05). Thus, to verify the robustness of the MR estimates and gain considerable confidence in the presented results, a replication analysis was conducted using two independent datasets, taken from the ILAE and FinnGen. The authors of the present study argue that a conservative threshold of multiple testing may obscure the associations that were potentially noteworthy when studied individually. Therefore, a suggestive threshold of p < 0.05 was used to identify potential candidate antihyperglycemic drug targets associated with epilepsy, and replication analyses were conducted using two independent datasets from the ILAE and FinnGen to validate the results. However, the authors acknowledge that the unique genetic profile of the Finnish population may introduce bias. Finally, although MR methods are excellent for causal inference, the results of this study should be confirmed in well-designed randomised controlled trials to establish a causal relationship.

## Conclusion

This MR study suggests that the antihyperglycemic drug target gene ETFDH may increase the risk of epilepsy and that metformin is an inhibitor of the ETFDH gene. This explains the potential therapeutic significance of metformin in the treatment of epilepsy and lays the groundwork for further mechanistic research.

### Electronic supplementary material

Below is the link to the electronic supplementary material.

Supplementary Material 1

## Data Availability

Data from DrugBank database (http://www.drugbank.ca/) and FinnGen consortium (http://r6.finngen.fi/) are publicly available. The corresponding author has full access to all data and material and can provide availability if needed.
