# Supplementary material for: Genetic prediction of antihyperglycemic drug targets and risk of epilepsy: a mendelian randomisation study
Source: BMC Pharmacol Toxicol. 2024 Jan 2;25:1. doi: 10.1186/s40360-023-00718-2 (PMC10763459; doi:10.1186/s40360-023-00718-2)
Supplement: Supplementary file 1 — Supplementary Material 1 [file 40360_2023_718_MOESM1_ESM.docx]

**Table S1.** The names of 74 drugs and information on 96 drug target genes

| **Name** | **Drugbank ID** | **Target genes** | **Ensembl ID** |
| --- | --- | --- | --- |
| Ebselen | DB12610 | EPHX2 | ENSG00000120915 |
| INCB13739 | DB05064 | HSD11B1 | ENSG00000117594 |
| PSN357 | DB05044 | PYGL | ENSG00000100504 |
| Bisegliptin | DB06127 | DPP4 | ENSG00000197635 |
| NOX‑700 | DB05464 | NFKB2 | ENSG00000077150 |
|  |  | NFKB1 | ENSG00000109320 |
| CLX‑0921 | DB05854 | PPARG | ENSG00000132170 |
| Reglitazar | DB04971 | PPARA | ENSG00000186951 |
|  |  | PPARG | ENSG00000132170 |
| ISIS 113715 | DB05506 | PTPN1 | ENSG00000196396 |
| AT1391 | DB05120 | INSR | ENSG00000171105 |
| NN344 | DB05115 | INSR | ENSG00000171105 |
|  |  | CYP1A2 | ENSG00000140505 |
| APD668 | DB05166 | GPR119 | ENSG00000147262 |
| Dutogliptin | DB11723 | DPP4 | ENSG00000197635 |
| MB‑07803 | DB05053 | FBP1 | ENSG00000165140 |
| PSN9301 | DB05001 | DPP4 | ENSG00000197635 |
| Gliquidone | DB01251 | ABCC8 | ENSG00000006071 |
|  |  | KCNJ8 | ENSG00000121361 |
|  |  | CYP2C9 | ENSG00000138109 |
| Albiglutide | DB09043 | GLP1R | ENSG00000112164 |
| Pramlintide | DB01278 | CALCR | ENSG00000004948 |
|  |  | RAMP1 | ENSG00000132329 |
|  |  | RAMP2 | ENSG00000131477 |
|  |  | RAMP3 | ENSG00000122679 |
| Voglibose | DB04878 | MGAM | ENSG00000257335 |
| Dapagliflozin | DB06292 | SLC5A2 | ENSG00000140675 |
|  |  | CYP1A1 | ENSG00000140465 |
|  |  | CYP1A2 | ENSG00000140505 |
|  |  | CYP2A6 | ENSG00000255974 |
|  |  | CYP2C9 | ENSG00000138109 |
|  |  | CYP2D6 | ENSG00000100197 |
|  |  | CYP3A4 | ENSG00000160868 |
|  |  | UGT1A9 | ENSG00000241119 |
|  |  | UGT2B4 | ENSG00000156096 |
|  |  | UGT2B7 | ENSG00000171234 |
| Miglitol | DB00491 | MGAM | ENSG00000257335 |
|  |  | GAA | ENSG00000171298 |
|  |  | GANAB | ENSG00000089597 |
|  |  | GANC | ENSG00000214013 |
|  |  | AMY2A | ENSG00000243480 |
| Vildagliptin | DB04876 | DPP4 | ENSG00000197635 |
| Dulaglutide | DB09045 | GLP1R | ENSG00000112164 |
| Phenformin | DB00914 | PRKAA1 | ENSG00000132356 |
|  |  | KCNJ8 | ENSG00000121361 |
|  |  | CYP2D6 | ENSG00000100197 |
| AMG‑131 | DB05490 | PPARG | ENSG00000132170 |
| Acarbose | DB00284 | MGAM | ENSG00000257335 |
|  |  | GAA | ENSG00000171298 |
|  |  | SI | ENSG00000090402 |
|  |  | AMY2A | ENSG00000243480 |
| Sitagliptin | DB01261 | DPP4 | ENSG00000197635 |
|  |  | CYP3A4 | ENSG00000160868 |
|  |  | CYP2C8 | ENSG00000138115 |
| Acetohexamide | DB00414 | KCNJ1 | ENSG00000151704 |
|  |  | CBR1 | ENSG00000159228 |
|  |  | CYP2C9 | ENSG00000138109 |
| Canagliflozin | DB08907 | SLC5A2 | ENSG00000140675 |
|  |  | UGT1A9 | ENSG00000241119 |
|  |  | UGT2B4 | ENSG00000156096 |
|  |  | CYP3A4 | ENSG00000160868 |
| Pioglitazone | DB01132 | PPARG | ENSG00000132170 |
|  |  | MAOB | ENSG00000069535 |
|  |  | CYP2C8 | ENSG00000138115 |
|  |  | CYP3A4 | ENSG00000160868 |
|  |  | CYP1A1 | ENSG00000140465 |
| Glisoxepide | DB01289 | KCNJ8 | ENSG00000121361 |
|  |  | CYP2C9 | ENSG00000138109 |
| Glipizide | DB01067 | ABCC8 | ENSG00000006071 |
|  |  | PPARG | ENSG00000132170 |
|  |  | CYP2C9 | ENSG00000138109 |
|  |  | UGT1A1 | ENSG00000241635 |
| Insulin Glargine | DB00047 | INSR | ENSG00000171105 |
|  |  | IGF1R | ENSG00000140443 |
|  |  | CYP1A2 | ENSG00000140505 |
| Insulin Degludec | DB09564 | INSR | ENSG00000171105 |
|  |  | IGF1R | ENSG00000140443 |
|  |  | CYP1A2 | ENSG00000140505 |
| Chlorpropamide | DB00672 | ABCC8 | ENSG00000006071 |
|  |  | CYP2C9 | ENSG00000138109 |
|  |  | CYP2C19 | ENSG00000165841 |
|  |  | PTGS1 | ENSG00000095303 |
| Linagliptin | DB08882 | DPP4 | ENSG00000197635 |
|  |  | CYP3A4 | ENSG00000160868 |
| Repaglinide | DB00912 | ABCC8 | ENSG00000006071 |
|  |  | PPARG | ENSG00000132170 |
|  |  | CYP2C8 | ENSG00000138115 |
|  |  | CYP3A4 | ENSG00000160868 |
| Insulin Pork | DB00071 | INSR | ENSG00000171105 |
|  |  | IGF1R | ENSG00000140443 |
|  |  | IDE | ENSG00000119912 |
|  |  | CYP1A2 | ENSG00000140505 |
| Nateglinide | DB00731 | ABCC8 | ENSG00000006071 |
|  |  | PPARG | ENSG00000132170 |
|  |  | CYP2C9 | ENSG00000138109 |
|  |  | CYP3A4 | ENSG00000160868 |
|  |  | CYP3A5 | ENSG00000106258 |
|  |  | CYP3A7 | ENSG00000160870 |
|  |  | PTGS1 | ENSG00000095303 |
|  |  | UGT1A9 | ENSG00000241119 |
|  |  | CYP2D6 | ENSG00000100197 |
| Insulin Aspart | DB01306 | INSR | ENSG00000171105 |
|  |  | IGF1R | ENSG00000140443 |
|  |  | CYP1A2 | ENSG00000140505 |
| Insulin Detemir | DB01307 | INSR | ENSG00000171105 |
|  |  | IGF1R | ENSG00000140443 |
|  |  | CYP1A2 | ENSG00000140505 |
| Saxagliptin | DB06335 | DPP4 | ENSG00000197635 |
|  |  | CYP3A4 | ENSG00000160868 |
|  |  | CYP3A5 | ENSG00000106258 |
| Insulin Glulisine | DB01309 | INSR | ENSG00000171105 |
|  |  | IGF1R | ENSG00000140443 |
|  |  | CYP1A2 | ENSG00000140505 |
| Tolbutamide | DB01124 | ABCC8 | ENSG00000006071 |
|  |  | KCNJ1 | ENSG00000151704 |
|  |  | CYP2C9 | ENSG00000138109 |
|  |  | CYP2C8 | ENSG00000138115 |
|  |  | CYP2C19 | ENSG00000165841 |
|  |  | CYP2C18 | ENSG00000108242 |
| Rosiglitazone | DB00412 | PPARG | ENSG00000132170 |
|  |  | ACSL4 | ENSG00000068366 |
|  |  | PPARA | ENSG00000186951 |
|  |  | PPARD | ENSG00000112033 |
|  |  | RXRA | ENSG00000186350 |
|  |  | RXRB | ENSG00000204231 |
|  |  | RXRG | ENSG00000143171 |
|  |  | CYP2C8 | ENSG00000138115 |
|  |  | CYP2C9 | ENSG00000138109 |
|  |  | PTGS1 | ENSG00000095303 |
|  |  | CYP1A2 | ENSG00000140505 |
|  |  | CYP3A4 | ENSG00000160868 |
|  |  | CYP2B6 | ENSG00000197408 |
|  |  | CYP2D6 | ENSG00000100197 |
|  |  | CYP2E1 | ENSG00000130649 |
| Mitiglinide | DB01252 | ABCC8 | ENSG00000006071 |
|  |  | PPARG | ENSG00000132170 |
|  |  | UGT1A3 | ENSG00000243135 |
|  |  | UGT2B7 | ENSG00000171234 |
| Insulin Human | DB00030 | INSR | ENSG00000171105 |
|  |  | IGF1R | ENSG00000140443 |
|  |  | CPE | ENSG00000109472 |
|  |  | NOV | NA |
|  |  | LRP2 | ENSG00000081479 |
|  |  | IGFBP7 | ENSG00000163453 |
|  |  | IDE | ENSG00000119912 |
|  |  | PCSK2 | ENSG00000125851 |
|  |  | PCSK1 | ENSG00000175426 |
|  |  | CYP1A2 | ENSG00000140505 |
| Insulin Lispro | DB00046 | INSR | ENSG00000171105 |
|  |  | IGF1R | ENSG00000140443 |
|  |  | CYP1A2 | ENSG00000140505 |
|  |  | IDE | ENSG00000119912 |
| Lixisenatide | DB09265 | GLP1R | ENSG00000112164 |
| Metformin | DB00331 | PRKAB1 | ENSG00000111725 |
|  |  | ETFDH | ENSG00000171503 |
|  |  | GPD1 | ENSG00000167588 |
| Lobeglitazone | DB09198 | PPARG | ENSG00000132170 |
|  |  | CYP1A2 | ENSG00000140505 |
|  |  | CYP2C9 | ENSG00000138109 |
|  |  | CYP2C19 | ENSG00000165841 |
|  |  | CYP3A4 | ENSG00000160868 |
| Managlinat dialanetil | DB05518 | FBP1 | ENSG00000165140 |
| Levoketoconazole | DB05667 | CYP11B1 | ENSG00000160882 |
|  |  | CYP51A1 | ENSG00000001630 |
|  |  | CYP3A4 | ENSG00000160868 |
|  |  | CYP3A5 | ENSG00000106258 |
|  |  | CYP51A1 | ENSG00000001630 |
|  |  | CYP17A1 | ENSG00000148795 |
|  |  | CYP21A2 | ENSG00000231852 |
|  |  | CYP11B1 | ENSG00000160882 |
| Tesaglitazar | DB06536 | PPARA | ENSG00000186951 |
|  |  | PPARG | ENSG00000132170 |
| Ertiprotafib | DB06521 | PTPN1 | ENSG00000196396 |
|  |  | IKBKB | ENSG00000104365 |
|  |  | PPARA | ENSG00000186951 |
|  |  | PPARG | ENSG00000132170 |
| Glycodiazine | DB01382 | KCNJ1 | ENSG00000151704 |
|  |  | ABCC8 | ENSG00000006071 |
| Muraglitazar | DB06510 | PPARA | ENSG00000186951 |
|  |  | PPARG | ENSG00000132170 |
|  |  | CYP1A2 | ENSG00000140505 |
|  |  | UGT1A3 | ENSG00000243135 |
|  |  | UGT1A1 | ENSG00000241635 |
|  |  | CYP2C8 | ENSG00000138115 |
| Troglitazone | DB00197 | PPARG | ENSG00000132170 |
|  |  | ACSL4 | ENSG00000068366 |
|  |  | SERPINE1 | ENSG00000106366 |
|  |  | SLC29A1 | ENSG00000112759 |
|  |  | ESRRG | ENSG00000196482 |
|  |  | ESRRA | NA |
|  |  | PPARD | ENSG00000112033 |
|  |  | PPARA | ENSG00000186951 |
|  |  | GSTP1 | ENSG00000084207 |
|  |  | CYP3A4 | ENSG00000160868 |
|  |  | CYP2C19 | ENSG00000165841 |
|  |  | UGT1A1 | ENSG00000241635 |
|  |  | CYP2C8 | ENSG00000138115 |
|  |  | CYP19A1 | ENSG00000137869 |
|  |  | CYP1A1 | ENSG00000140465 |
|  |  | CYP2B6 | Enzyme |
|  |  | CYP2C9 | ENSG00000138109 |
|  |  | CYP3A5 | ENSG00000106258 |
|  |  | CYP3A7 | ENSG00000160870 |
|  |  | UGT1A3 | ENSG00000243135 |
|  |  | UGT1A4 | ENSG00000244474 |
|  |  | UGT1A6 | ENSG00000167165 |
|  |  | UGT1A7 | ENSG00000244122 |
|  |  | UGT1A8 | ENSG00000242366 |
|  |  | UGT1A9 | ENSG00000241119 |
|  |  | UGT1A10 | ENSG00000242515 |
|  |  | UGT2B7 | ENSG00000171234 |
|  |  | UGT2B15 | ENSG00000277132 |
| Ertugliflozin | DB11827 | SLC5A2 | ENSG00000140675 |
|  |  | UGT1A9 | ENSG00000241119 |
|  |  | UGT2B7 | ENSG00000171234 |
|  |  | UGT1A1 | ENSG00000241635 |
|  |  | UGT1A4 | ENSG00000244474 |
| Exenatide | DB01276 | GLP1R | ENSG00000112164 |
|  |  | DPP4 | ENSG00000197635 |
| Naveglitazar | DB12662 | PPARG | ENSG00000132170 |
| Alogliptin | DB06203 | DPP4 | ENSG00000197635 |
|  |  | CYP3A4 | ENSG00000160868 |
|  |  | CYP2D6 | ENSG00000100197 |
| Liraglutide | DB06655 | GLP1R | ENSG00000112164 |
|  |  | DPP4 | ENSG00000197635 |
|  |  | MME | ENSG00000196549 |
| Semaglutide | DB13928 | GLP1R | ENSG00000112164 |
|  |  | DPP4 | ENSG00000197635 |
|  |  | MME | ENSG00000196549 |
|  |  | LPL | ENSG00000175445 |
|  |  | AMY1A | ENSG00000237763 |
| Glimepiride | DB00222 | KCNJ11 | ENSG00000187486 |
|  |  | KCNJ1 | ENSG00000151704 |
|  |  | ABCC8 | ENSG00000006071 |
|  |  | CYP2C9 | ENSG00000138109 |
| Sarpogrelate | DB12163 | HTR2C | ENSG00000147246 |
|  |  | HTR2A | ENSG00000102468 |
| Glyburide | DB01016 | ABCC9 | ENSG00000069431 |
|  |  | ABCB11 | ENSG00000073734 |
|  |  | ABCA1 | ENSG00000165029 |
|  |  | CFTR | ENSG00000001626 |
|  |  | CPT1A | ENSG00000110090 |
|  |  | TRPM4 | ENSG00000130529 |
|  |  | CYP2C9 | ENSG00000138109 |
|  |  | CYP2C19 | ENSG00000165841 |
|  |  | CYP3A4 | ENSG00000160868 |
|  |  | CYP3A7 | ENSG00000160870 |
|  |  | CYP3A5 | ENSG00000106258 |
|  | DB01120 | ABCC8 | ENSG00000006071 |
|  |  | VEGFA | ENSG00000112715 |
|  |  | CYP2C9 | ENSG00000138109 |
|  |  | CYP2C19 | ENSG00000165841 |
| Empagliflozin | DB09038 | SLC5A2 | ENSG00000140675 |
|  |  | UGT2B7 | ENSG00000171234 |
|  |  | UGT1A3 | ENSG00000243135 |
|  |  | UGT1A8 | ENSG00000242366 |
|  |  | UGT1A9 | ENSG00000241119 |
| Glymidine | DB01382 | KCNJ1 | ENSG00000151704 |
|  |  | ABCC8 | ENSG00000006071 |
| Balaglitazone | DB12781 | CYP3A4 | ENSG00000160868 |
|  |  | CYP2C8 | ENSG00000138115 |
| Glibornuride | DB08962 | CYP2C9 | ENSG00000138109 |
| Rivoglitazone | DB09200 | CYP3A4 | ENSG00000160868 |
|  |  | CYP2C8 | ENSG00000138115 |
| AB192 | DB06111 | MPO | ENSG00000005381 |
| Lisofylline | DB12406 | CYP1A2 | ENSG00000140505 |

**Table S2.** MR estimation of antiglycemic drug target genes with the International League Against Epilepsy GWAS during the preliminary analysis phase.

| Drug target | id.outcome | outcome | tissue | method | nsnp | b | se | pval |
| --- | --- | --- | --- | --- | --- | --- | --- | --- |
| CYP2E1 | ieu-b-8 | epilepsy | Brain_Anterior_cingulate_cortex | Wald ratio | 1 | -0.079855248 | 0.026147801 | 0.00225816 |
| CFTR | ieu-b-8 | epilepsy | Brain_Caudate_basal_ganglia | Inverse variance weighted | 2 | 0.079143397 | 0.027501063 | 0.004004203 |
| GAA | ieu-b-8 | epilepsy | Brain_Cerebellar_Hemisphere | Inverse variance weighted (fixed effects) | 5 | 0.017924063 | 0.006407333 | 0.005151101 |
| CYP2D6 | ieu-b-8 | epilepsy | Brain_Cortex | Inverse variance weighted (fixed effects) | 6 | -0.015492395 | 0.005566947 | 0.005387131 |
| MGAM | ieu-b-8 | epilepsy | Brain_Frontal_Cortex | Inverse variance weighted | 2 | -0.033189999 | 0.012156427 | 0.006328768 |
| CYP17A1 | ieu-b-8 | epilepsy | Brain_Cortex | Wald ratio | 1 | -0.040868245 | 0.015014033 | 0.006488751 |
| ETFDH | ieu-b-8 | epilepsy | Brain_Cerebellar_Hemisphere | Inverse variance weighted (fixed effects) | 7 | 0.018642276 | 0.007145262 | 0.009079654 |
| NFKB2 | ieu-b-8 | epilepsy | Brain_Nucleus_accumbens_basal_ganglia | Wald ratio | 1 | 0.109857736 | 0.042236687 | 0.009295173 |
| GAA | ieu-b-8 | epilepsy | Brain_Frontal_Cortex | Inverse variance weighted (fixed effects) | 4 | 0.028127937 | 0.011488759 | 0.014353179 |
| CYP21A2 | ieu-b-8 | epilepsy | Brain_Cerebellum | Inverse variance weighted | 2 | 0.028507761 | 0.011902962 | 0.016619733 |
| FBP1 | ieu-b-8 | epilepsy | Brain_Hypothalamus | Wald ratio | 1 | 0.030394679 | 0.012706828 | 0.01675703 |
| CYP3A5 | ieu-b-8 | epilepsy | Brain_Nucleus_accumbens_basal_ganglia | Inverse variance weighted | 2 | -0.037077993 | 0.015735013 | 0.018453006 |
| HTR2A | ieu-b-8 | epilepsy | Brain_Caudate_basal_ganglia | Wald ratio | 1 | -0.055011716 | 0.023349593 | 0.018472758 |
| SLC5A2 | ieu-b-8 | epilepsy | Brain_Cerebellar_Hemisphere | Wald ratio | 1 | -0.053197137 | 0.022782439 | 0.019543097 |
| ABCC8 | ieu-b-8 | epilepsy | Brain_Anterior_cingulate_cortex | Inverse variance weighted (fixed effects) | 3 | 0.041078161 | 0.017667724 | 0.020069829 |
| MGAM | ieu-b-8 | epilepsy | Brain_Caudate_basal_ganglia | Wald ratio | 1 | -0.04242295 | 0.018729773 | 0.023512632 |
| CYP2D6 | ieu-b-8 | epilepsy | Brain_Anterior_cingulate_cortex | Inverse variance weighted (fixed effects) | 4 | -0.01660883 | 0.007426274 | 0.025319323 |
| CYP2E1 | ieu-b-8 | epilepsy | Brain_Cortex | Wald ratio | 1 | 0.034699016 | 0.015539214 | 0.025549157 |
| CYP17A1 | ieu-b-8 | epilepsy | Brain_Frontal_Cortex | Wald ratio | 1 | -0.042643412 | 0.019392158 | 0.027877712 |
| GAA | ieu-b-8 | epilepsy | Brain_Cortex | Inverse variance weighted (fixed effects) | 6 | 0.016693594 | 0.00760106 | 0.02807626 |
| CYP2D6 | ieu-b-8 | epilepsy | Brain_Nucleus_accumbens_basal_ganglia | Inverse variance weighted (fixed effects) | 4 | -0.017242958 | 0.007852281 | 0.028097872 |
| GAA | ieu-b-8 | epilepsy | Brain_Amygdala | Inverse variance weighted | 2 | 0.026280734 | 0.012045202 | 0.029121158 |
| IGF1R | ieu-b-8 | epilepsy | Brain_Cerebellar_Hemisphere | Wald ratio | 1 | -0.0563649 | 0.026010552 | 0.030234754 |
| CYP2E1 | ieu-b-8 | epilepsy | Brain_Caudate_basal_ganglia | Wald ratio | 1 | 0.051453394 | 0.024201978 | 0.033503299 |
| KCNJ11 | ieu-b-8 | epilepsy | Brain_Caudate_basal_ganglia | Wald ratio | 1 | 0.042721232 | 0.020142102 | 0.0339225 |
| MGAM | ieu-b-8 | epilepsy | Brain_Putamen_basal_ganglia | Wald ratio | 1 | -0.041252116 | 0.019569307 | 0.035030916 |
| CYP2D6 | ieu-b-8 | epilepsy | Brain_Cerebellum | Inverse variance weighted (fixed effects) | 8 | -0.009752104 | 0.004791349 | 0.041815024 |
| LPL | ieu-b-8 | epilepsy | Brain_Cerebellum | Wald ratio | 1 | -0.078744183 | 0.038905217 | 0.04297009 |
| CYP2D6 | ieu-b-8 | epilepsy | Brain_Caudate_basal_ganglia | Inverse variance weighted (fixed effects) | 3 | -0.027489153 | 0.013629994 | 0.043714995 |
| MGAM | ieu-b-8 | epilepsy | Brain_Cerebellar_Hemisphere | Inverse variance weighted | 2 | -0.021391146 | 0.010662218 | 0.044828871 |
| CYP3A5 | ieu-b-8 | epilepsy | Brain_Cerebellum | Inverse variance weighted (fixed effects) | 4 | -0.02706016 | 0.013490931 | 0.044877189 |
| GAA | ieu-b-8 | epilepsy | Brain_Cerebellum | Inverse variance weighted (fixed effects) | 5 | 0.010929471 | 0.00546373 | 0.04546055 |
| PPARG | ieu-b-8 | epilepsy | Brain_Cerebellum | Inverse variance weighted (fixed effects) | 7 | -0.009173489 | 0.004635833 | 0.047836032 |
| CYP2D6 | ieu-b-8 | epilepsy | Brain_Spinal_cord_cervical | Inverse variance weighted | 2 | -0.013564195 | 0.006886384 | 0.048871344 |

Note: “Drug target” is the exposure in the MR study, “epilepsy” is the outcome in the MR study, “tissue” is the location of antiglycemic drug target gene expression, “nsnp” refers to the number of instrumental variables used, “method” refers to the meta method we used, “beta” refers to the effect size in MR analysis, “se” refers to the standard error in MR analysis, and “P” is the statistical significance of MR analysis.


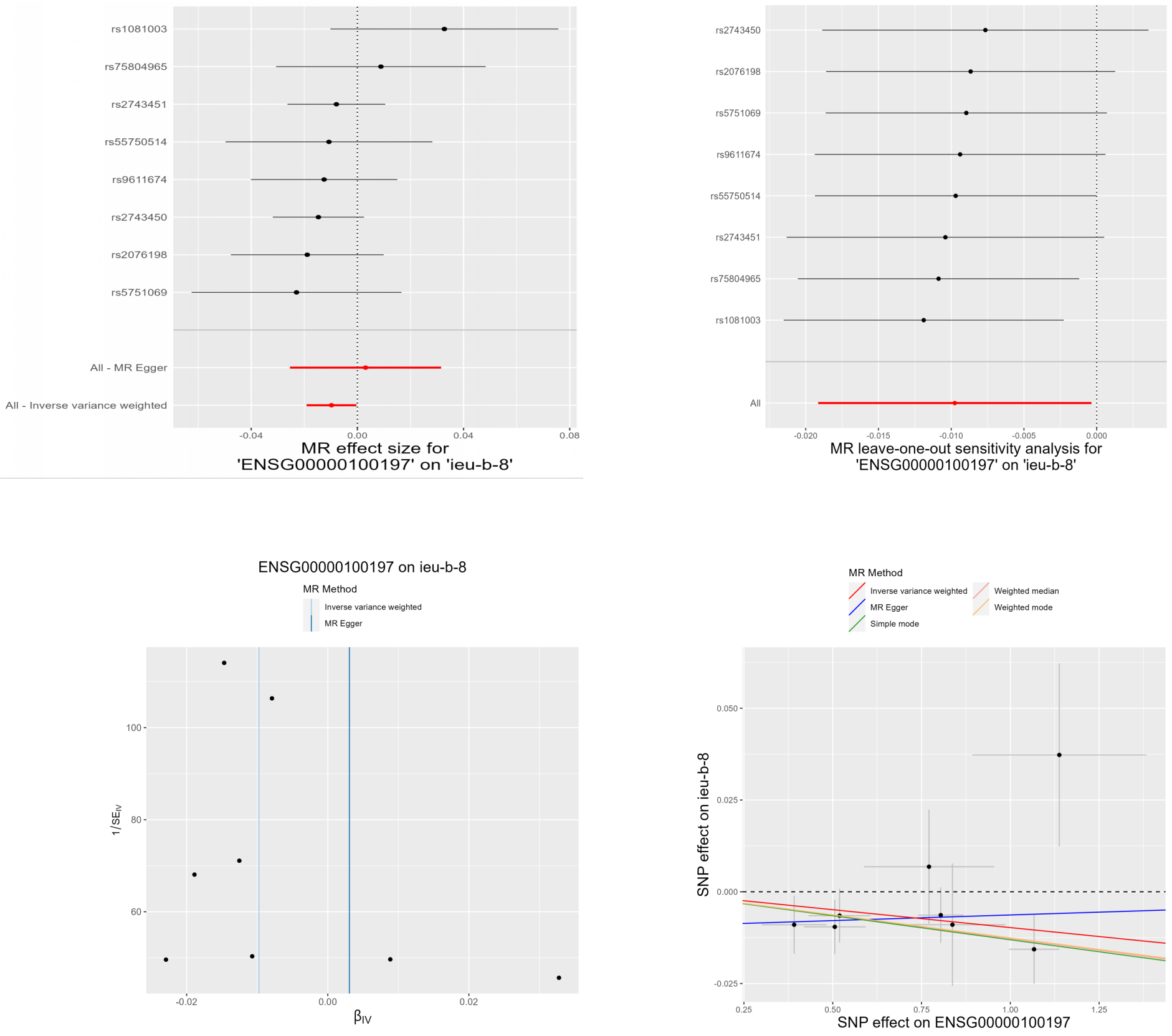


**Figure S1.** Mendelian randomization assessment of CYP2D6 expression in the brain-cerebellum and epilepsy risk.


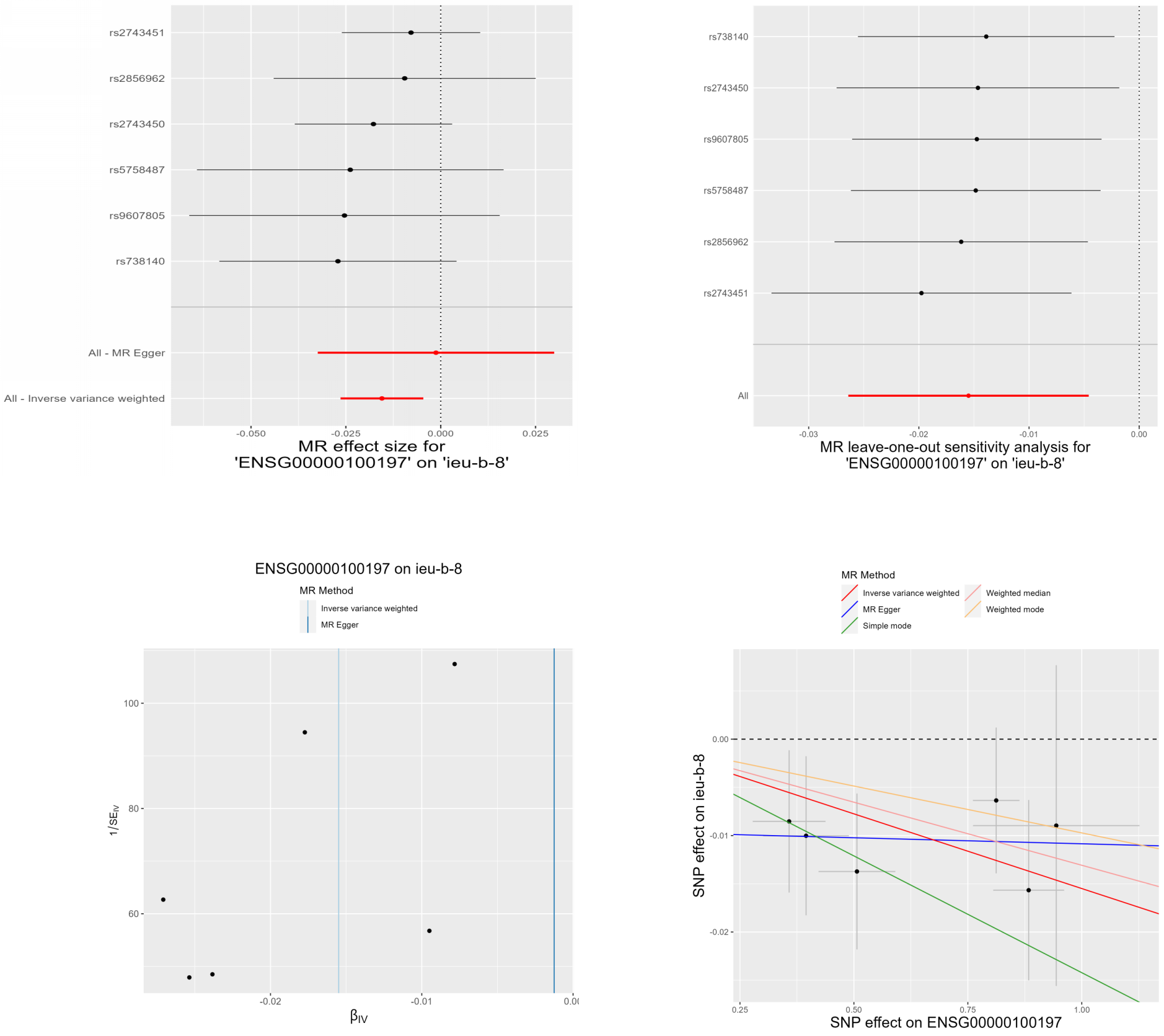


**Figure S2.** Mendelian randomization assessment of CYP2D6 expression in the brain-cortex and epilepsy risk.


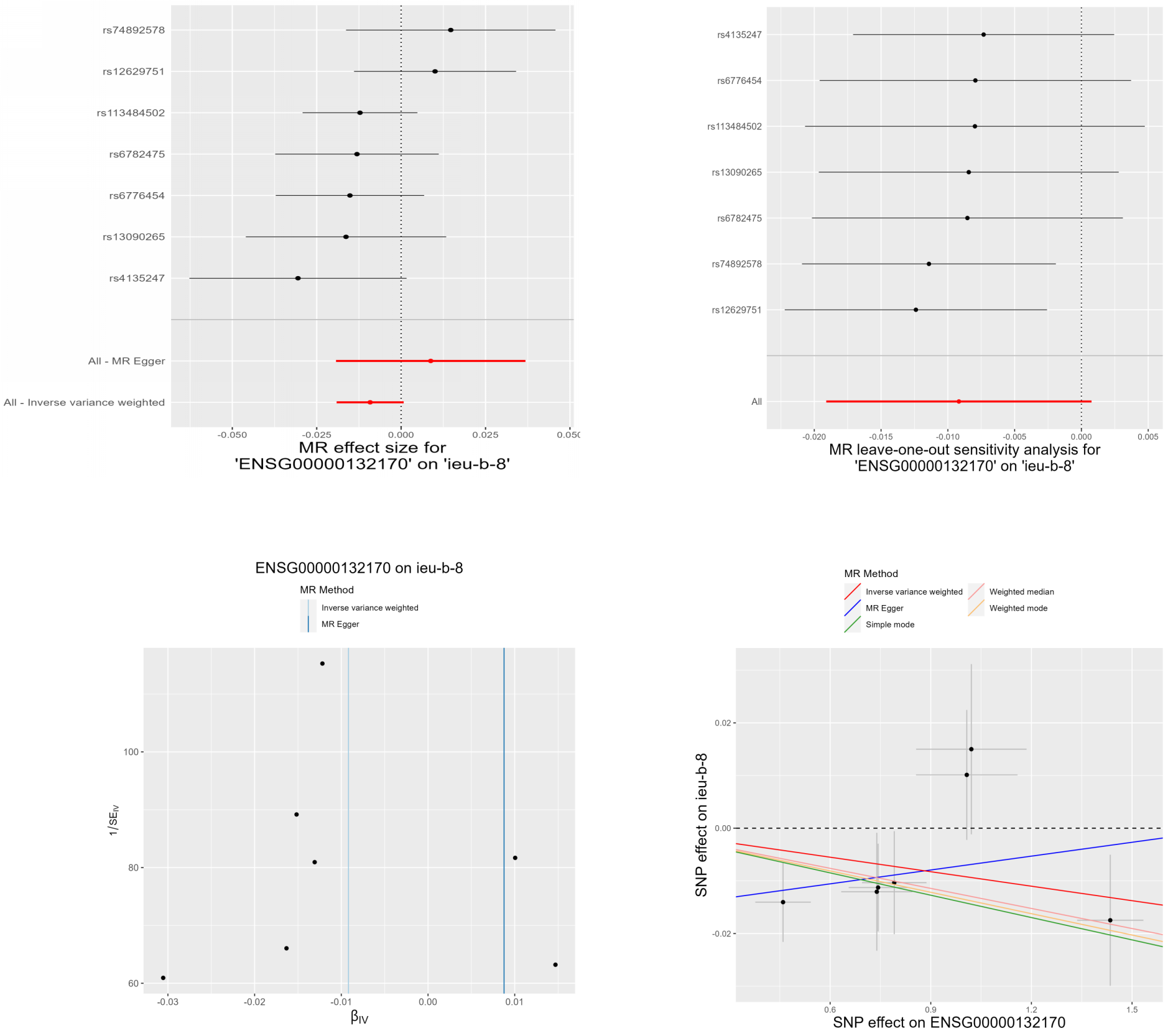


**Figure S3.** Mendelian randomization assessment of PPARG expression in the brain-cerebellum and epilepsy risk.


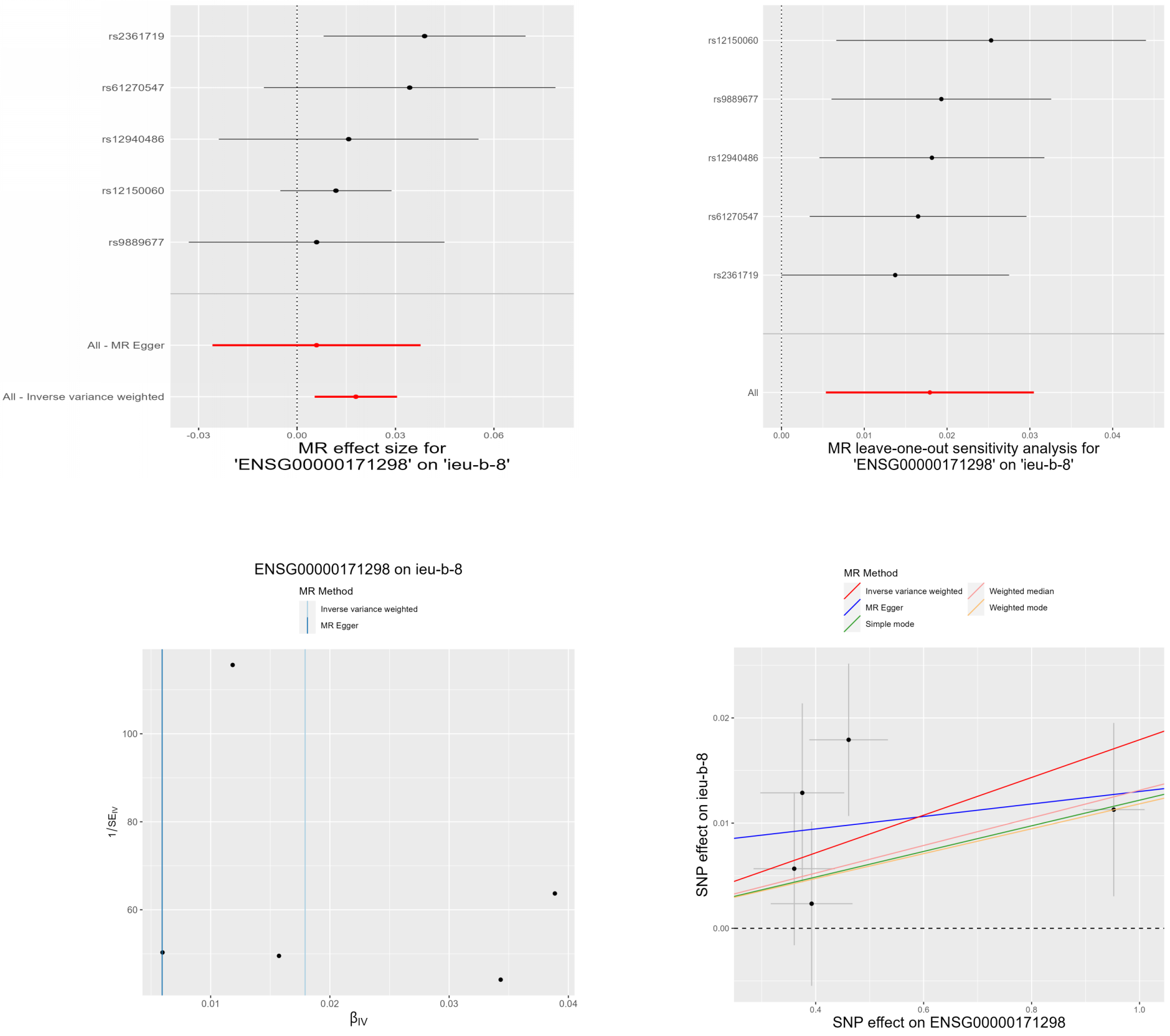


**Figure S4.** Mendelian randomization assessment of GAA expression in the brain-cerebellar-hemisphere and epilepsy risk.


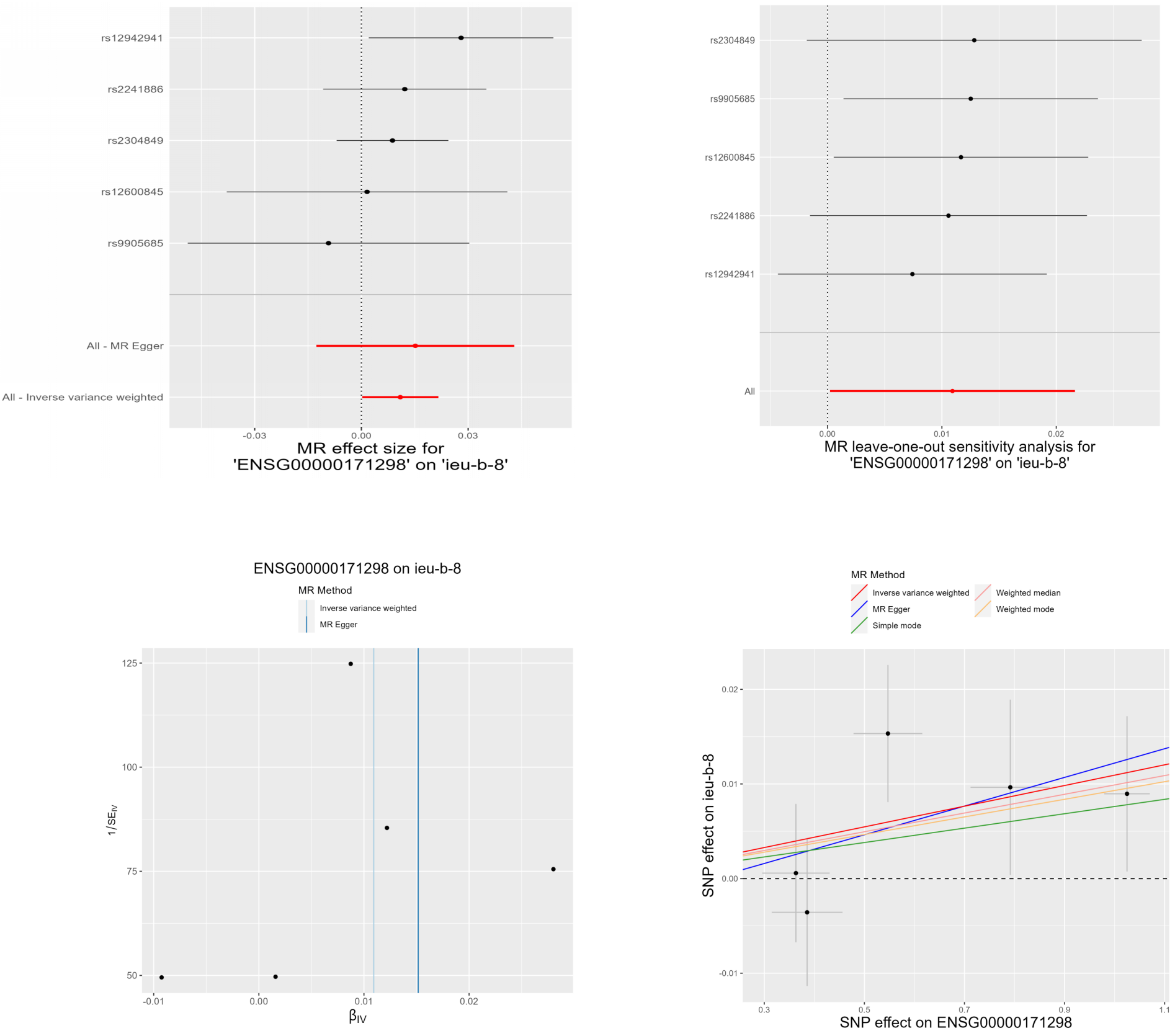


**Figure S5.** Mendelian randomization assessment of GAA expression in the brain-cerebellum and epilepsy risk.


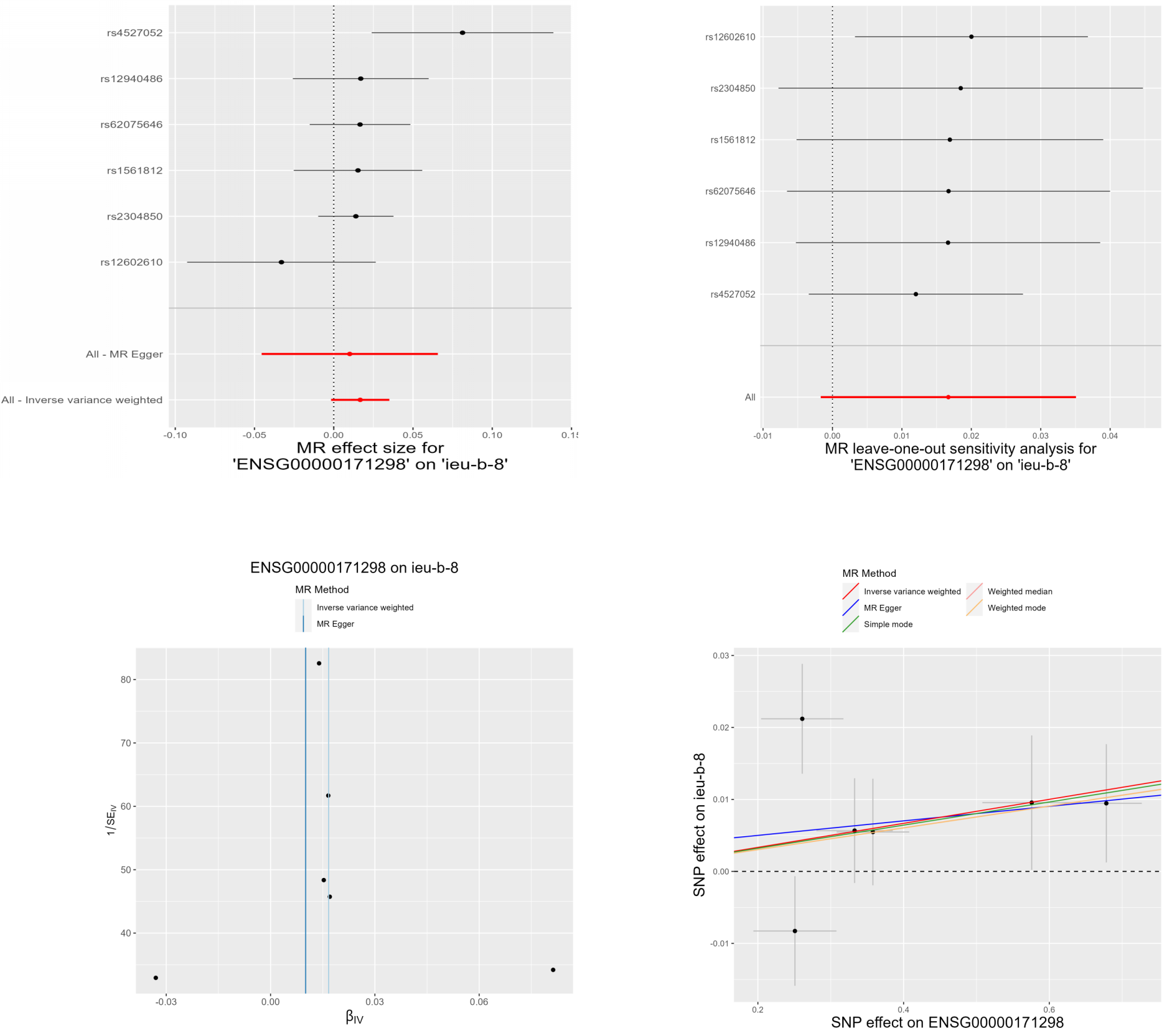


**Figure S6.** Mendelian randomization assessment of GAA expression in the brain-cerebellum and epilepsy risk.


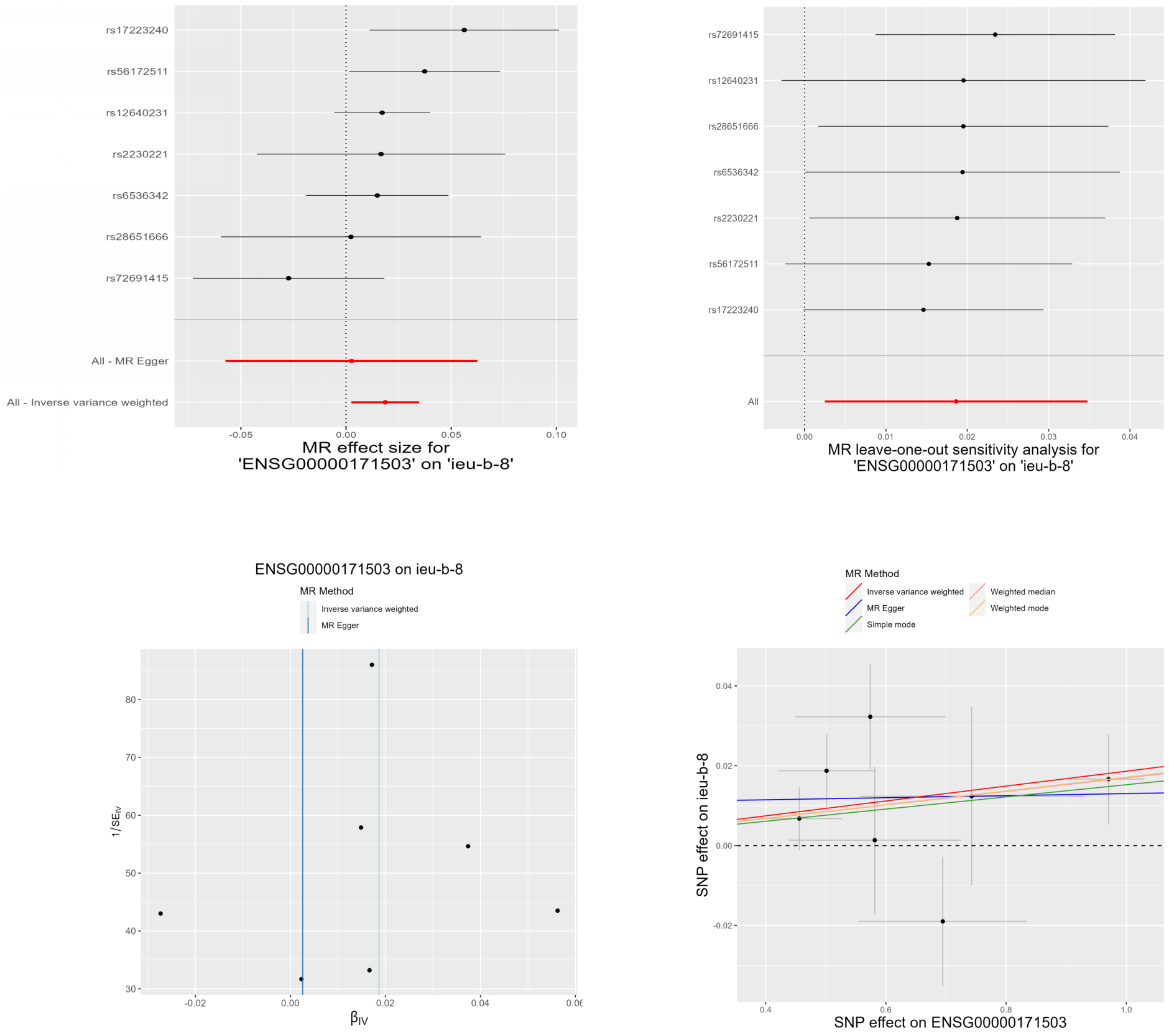


**Figure S7.** Mendelian randomization assessment of ETFDH expression in the brain-cerebellar-hemisphere and epilepsy risk.


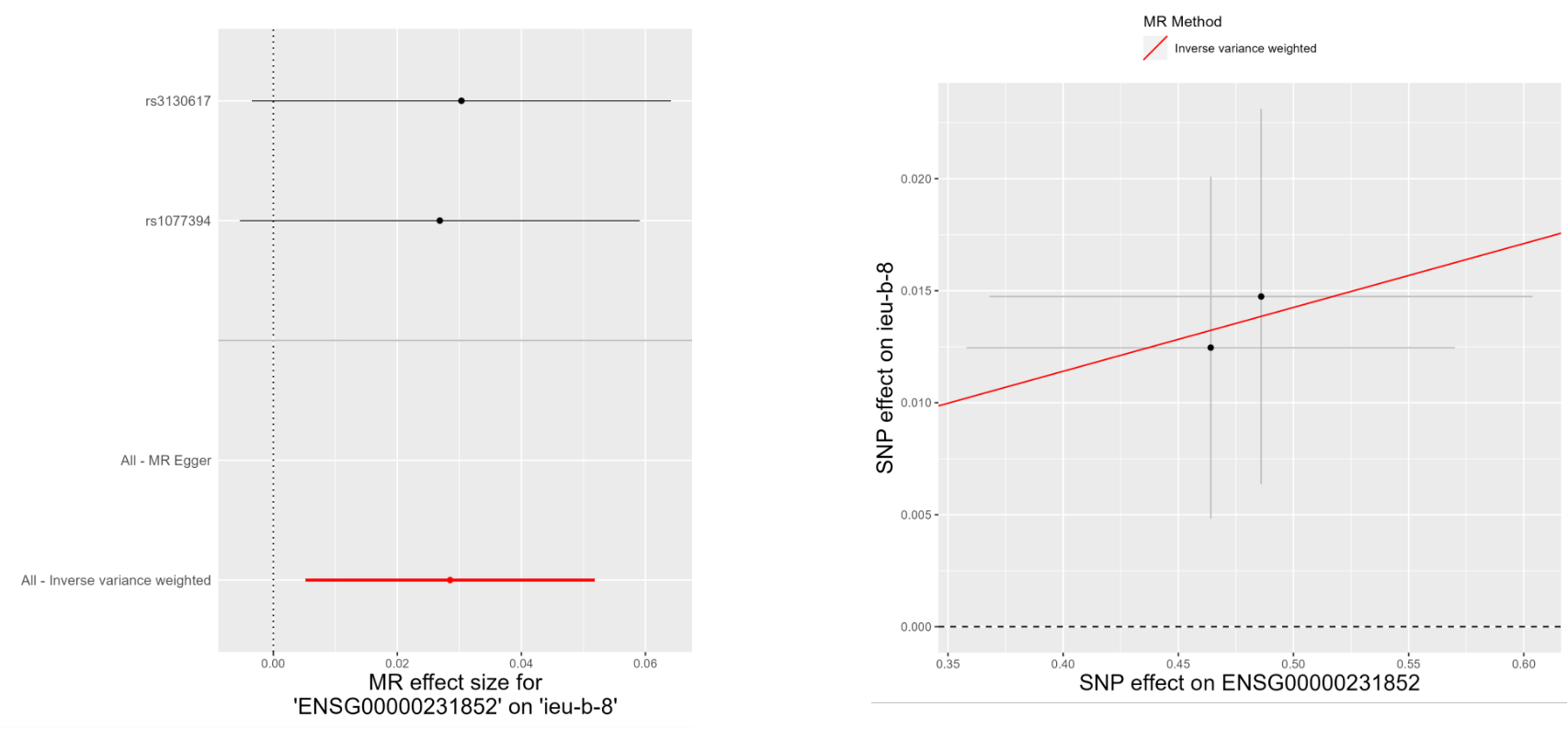


**Figure S8.** Mendelian randomization assessment of the CYP21A2 expression in brain_cerebellum and epilepsy risk in ILAE.


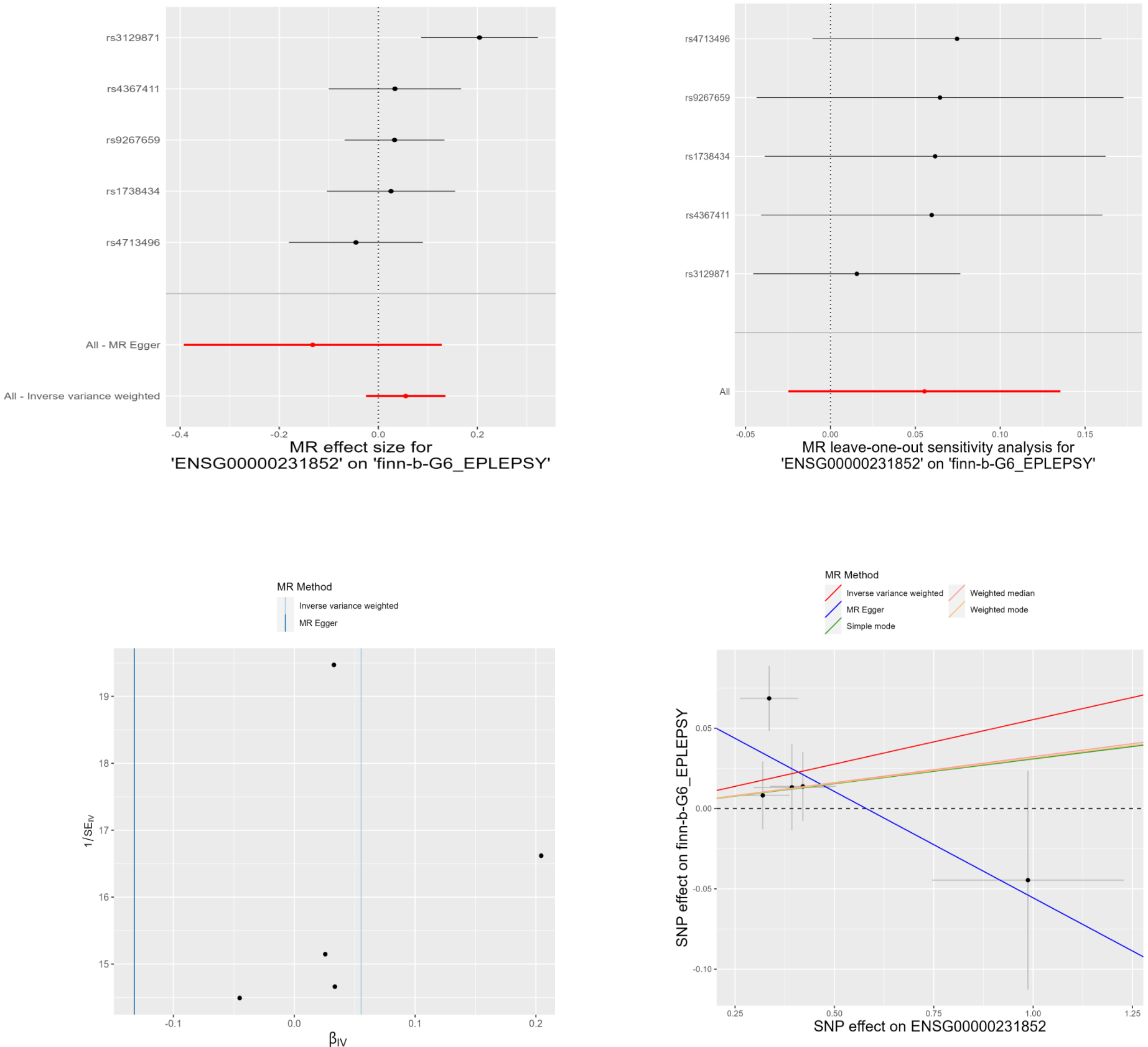


**Figure S9.** Mendelian randomization assessment of the CYP21A2 expression in brain_nucleus_accumbens_basal_ganglia and epilepsy risk in Finn Gen.


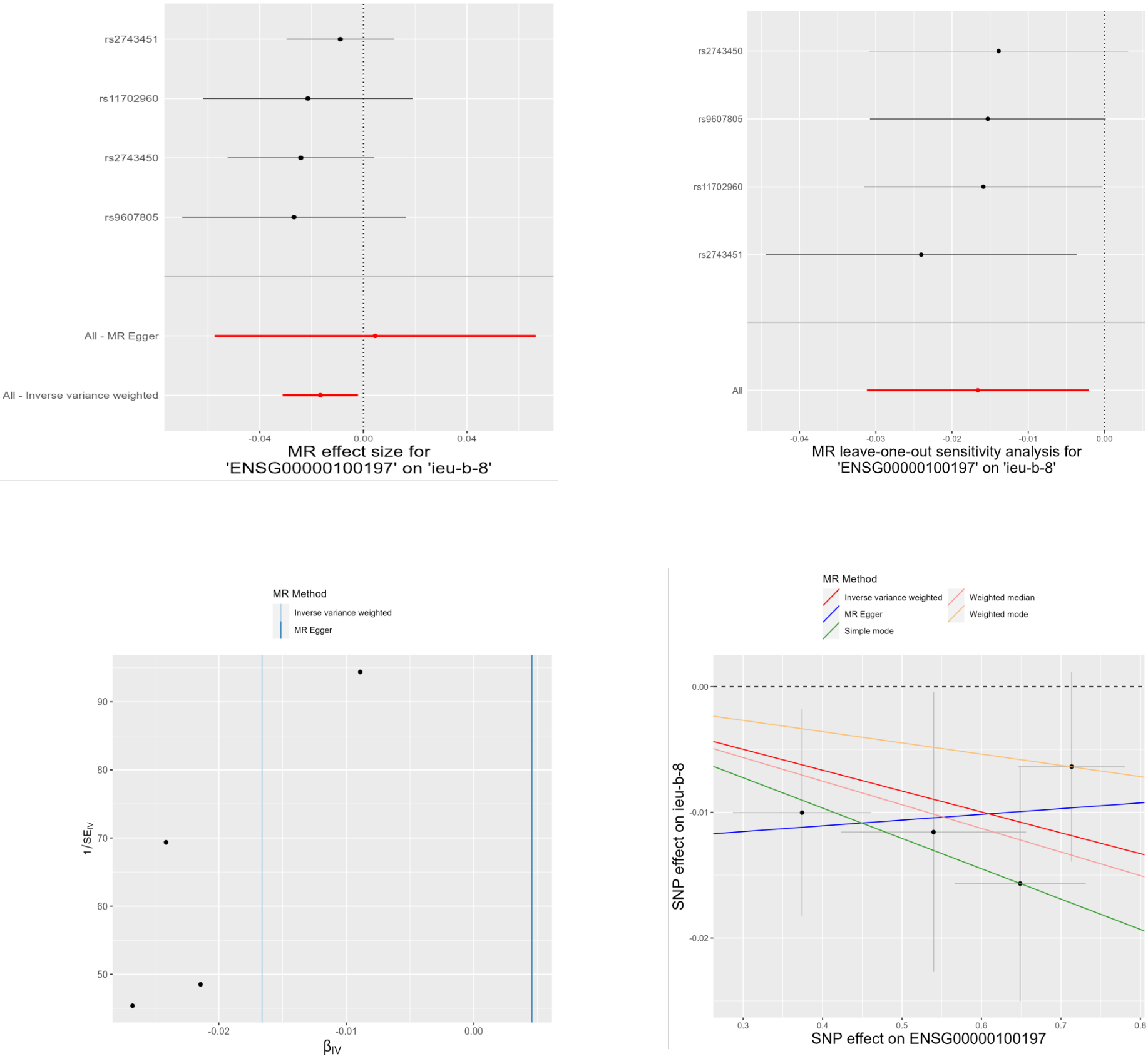


**Figure S10.** Mendelian randomization assessment of the CYP2D6 expression in brain_anterior_cingulate_cortex and epilepsy risk in ILAE.


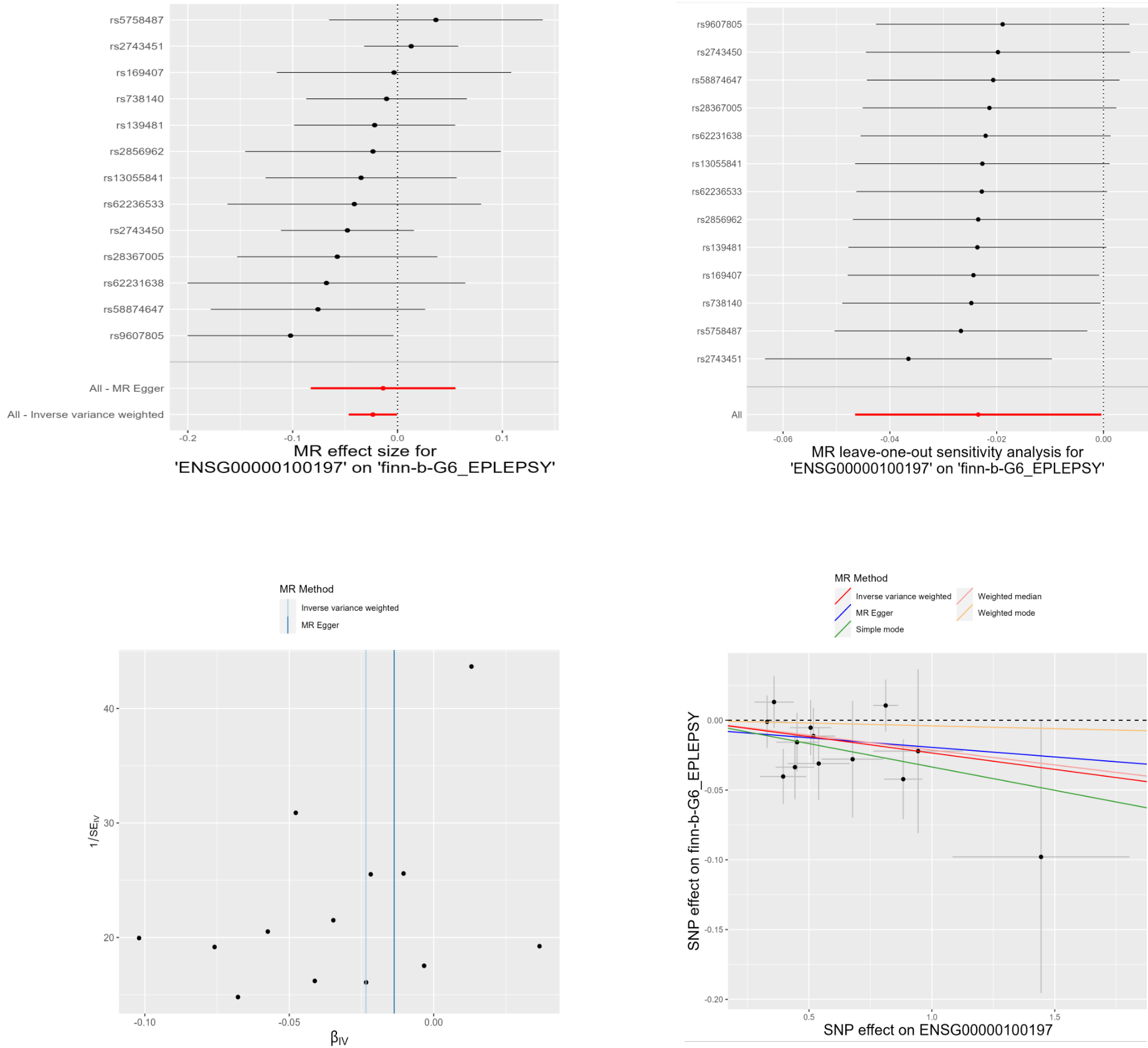


**Figure S11 .** Mendelian randomization assessment of the CYP2D6 expression in brain_cortex and epilepsy risk in Finn Gen.
